# *In vitro* angiogenesis in response to biomaterial properties for bone tissue engineering: a review of the state of the art

**DOI:** 10.1093/rb/rbad027

**Published:** 2023-03-27

**Authors:** Else Ellermann, Nima Meyer, Ruth E Cameron, Serena M Best

**Affiliations:** Department of Materials Science and Metallurgy, University of Cambridge, Cambridge CB3 0FS, UK; Department of Materials Science and Metallurgy, University of Cambridge, Cambridge CB3 0FS, UK; Department of Materials Science and Metallurgy, University of Cambridge, Cambridge CB3 0FS, UK; Department of Materials Science and Metallurgy, University of Cambridge, Cambridge CB3 0FS, UK

**Keywords:** angiogenesis, collagen scaffolds, hydroxyapatite, co-culture, architectural features, physicochemical properties

## Abstract

Bone tissue engineering (BTE) aims to improve the healing of bone fractures using scaffolds that mimic the native extracellular matrix. For successful bone regeneration, scaffolds should promote simultaneous bone tissue formation and blood vessel growth for nutrient and waste exchange. However, a significant challenge in regenerative medicine remains the development of grafts that can be vascularized successfully. Amongst other things, optimization of physicochemical conditions of scaffolds is key to achieving appropriate angiogenesis in the period immediately following implantation. Calcium phosphates and collagen scaffolds are two of the most widely studied biomaterials for BTE, due to their close resemblance to inorganic and organic components of bone, respectively, and their bioactivity, tunable biodegradability and the ability to produce tailored architectures. While various strategies exist to enhance vascularization of these scaffolds *in vivo*, further *in vitro* assessment is crucial to understand the relation between physicochemical properties of a biomaterial and its ability to induce angiogenesis. While mono-culture studies can provide evidence regarding cell–material interaction of a single cell type, a co-culture procedure is crucial for assessing the complex mechanisms involved in angiogenesis. A co-culture more closely resembles the natural tissue both physically and biologically by stimulating natural intercellular interactions and mimicking the organization of the *in vivo* environment. Nevertheless, a co-culture is a complex system requiring optimization of various parameters including cell types, cell ratio, culture medium and seeding logistics. Gaining fundamental knowledge of the mechanism behind the bioactivity of biomaterials and understanding the contribution of surface and architectural features to the vascularization of scaffolds, and the biological response in general, can provide an invaluable basis for future optimization studies. This review gives an overview of the available literature on scaffolds for BTE, and trends are extracted on the relationship between architectural features, biochemical properties, co-culture parameters and angiogenesis.

## Graphical abstract

**Figure:**
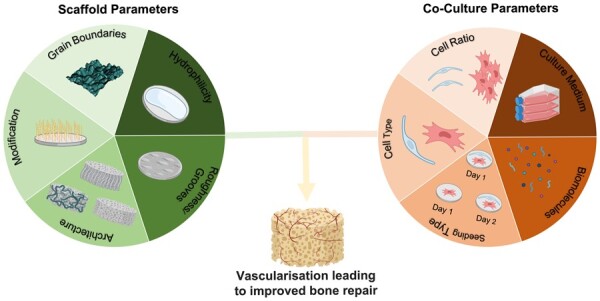

## Introduction

Healthy bone exhibits exceptional bone remodelling and regenerative properties. After injury, the structure can be restored to its natural pathology, provided there is a supply of viable cells, adequate vascularization for nutrient and waste exchange, stability, growth factors, and a matrix to allow cell attachment and proliferation. However, depending on the type of injury one or more of these conditions might not be met and osteosynthesis may be impeded [1, 2]. The density, stability and overall quality of the bone reduce with age and there can be an increased risk of bone fracture and reduced restorative capacity due to osteoporosis [3, 4]. In the UK alone, the proportion of the population over 65 years of age was estimated at 15.8% in 1999 which increased to 18.5% in 2019 and is expected to increase further to 23.9% by 2039, whilst the population under 16 is expected to decline from 20.4% in 1999to 16.9% by 2039 [5]. According to a study carried out in France, Germany, Italy, Spain, the UK and Sweden together, the fragility fracture incidence in 2017, responsible for an annual cost of ∼€37.5 billion, is expected to increase by 23% by 2030 due to the ageing society, accounting for an estimated annual cost of €47.6 billion. Even more striking is the increase of 25.6% in the total number of quality-adjusted life years lost by 2030 compared with 2017, highlighting the demand for improved treatment options [6, 7]. Currently, the main treatment option for critical-sized bone defects involves bone grafting.

Bone grafting augments natural healing by providing an osteoconductive, osteoinductive and/or osteogenic environment. Osteoconduction is the process of passively accommodating cells needed for bone tissue regeneration. Osteoinduction involves the recruitment, proliferation and differentiation of host mesenchymal stem cells (MSCs). Furthermore, a scaffold is considered osteogenic when all the cellular elements, growth factors (GFs) and scaffolding required to form new bone are available, see definitions in Bone Grafts and their Substitutes, Fillingham [1, 2]. To date, autografting is considered the golden standard procedure. An autograft is harvested from a different site in the patient’s body, often the posterior iliac crest, and it, therefore, provides an osteoconductive and osteoinductive environment and contains living osteogenic cells. However, this treatment requires an additional surgical procedure, it is associated with a high donor site morbidity, and, as can be expected, there is a limited supply. Allografting, which involves bone harvested from same-species donors, overcomes the need for additional surgery and is not associated with donor site morbidity. However, as human tissue is often not available in sufficient quantity to meet the increasing need for bone grafts, there is a need for alternative treatment, which resulted in a wide interest in bone tissue engineering (BTE) [1, 2].

**Figure 1.**
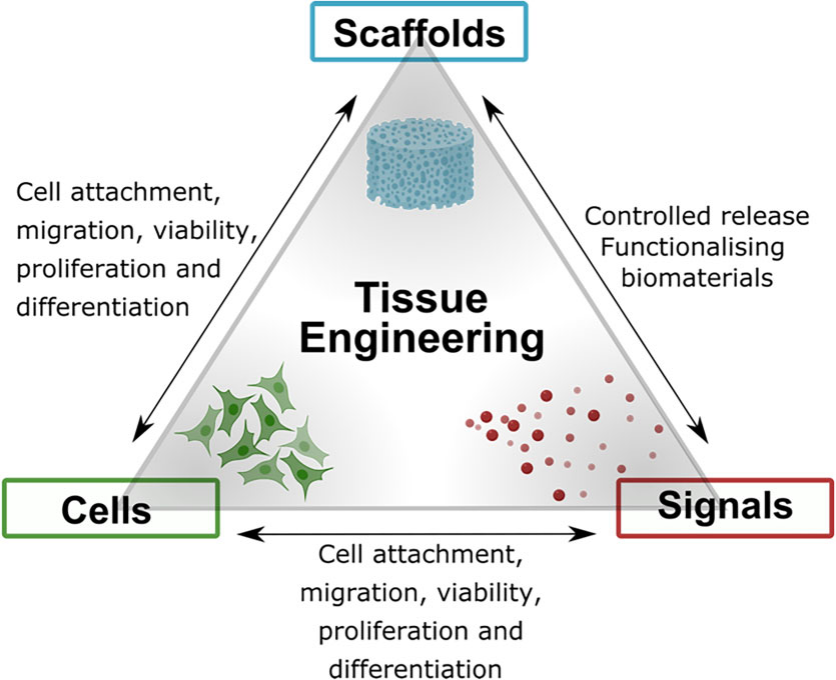
Schematic illustrating the necessary elements for tissue engineering, including tissue-specific cells, scaffolds acting as a template for cell migration, proliferation and tissue formation, and biomolecules providing the necessary signals for the optimal cell response.

BTE aims to regenerate bone tissue to its original healthy state, by using a temporary biomaterial. Achieving the optimum host response requires a synergistic interaction between a scaffold, cells and biomolecules, which are the main components involved in BTE as shown in Fig. 1. Scaffolds provide a porous 3D structure, mimicking the ECM and define the shape and form of the regenerating tissue. These scaffolds accommodate cells which can be differentiated into the desired cell type through the careful fine-tuning of various scaffold features. In addition, biomolecules such as growth factors (GFs) can be used to induce a specific cellular activity [8, 9].

**Figure 2.**
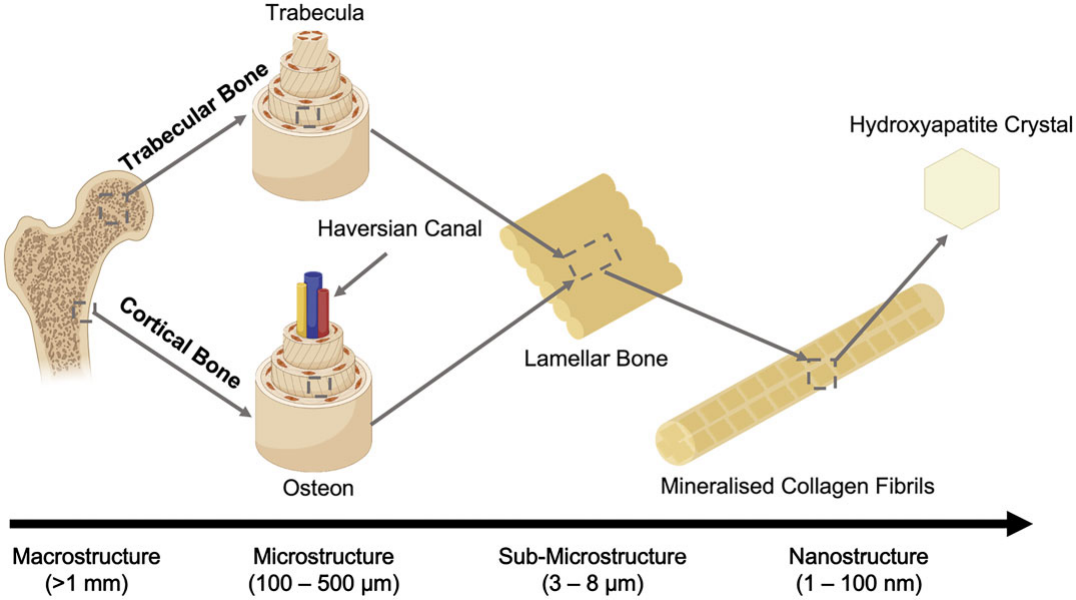
Hierarchical organization of bone. Adapted and reproduced from Ref. [12]. Copyright ©2012, Elsevier Ltd.

For successful implementation of BTE, it is crucial to understand the complex hierarchical structure of bone and its vascular network. Mineralized collagen fibrils are the ‘building blocks’ of bone tissue and consist of an organic phase containing collagen, proteoglycans and proteins, and an inorganic phase similar in composition to carbonated apatite (*Ca*_10_(*PO*_4_, *CO*_3_)_6_(*OH*)_2_) [10]. The inorganic component of bone consists of aligned nanosized, elongated platelet-like carbonated calcium phosphate particles. While bone mineral is similar in composition to stoichiometric hydroxyapatite (HA), it differs from this material due to its inherent disorder and nonstoichiometric properties as a result of the presence of multiple ionic species, such as ${\text{CO}}_{3}^{2-}$, Na^+^, Mg^2+^ and Si^4+^, and ion vacancies. Each of the ionic substitutions, even in very low amounts, is believed to play a significant role in bone metabolism although many of the mechanisms are still poorly understood [11]. As seen in Fig. 2, the organic and inorganic phases associate with each other to form lamellae, creating dense cortical bone and porous trabecular bone [12]. The cellular component of bone is responsible for only 2% of the total body mass but plays a key role in bone metabolic function, remodelling and regeneration [13]. The different types of cells involved include osteoblasts (OBs), bone lining cells, osteocytes and osteoclasts. OBs can secrete a non-mineralized osteoid matrix essential for healthy bone remodelling, and once embedded in the calcified matrix, OBs become osteocytes. Osteocytes produce a network responsible for nutrient and waste transfer and are the primary mechanosensors in bone. Bone lining cells play an essential role in communication through gap junctions with osteocytes, promoting hematopoietic stem cell differentiation into osteoclasts that are responsible for bone resorption [13]. Lastly, an intraosseous vasculature network is responsible for the nutrient and waste exchange within the bone and is, therefore, crucial for cell survival [14]. Endothelial cells (ECs) are responsible for the formation of a vascular network. The majority of the blood vessels in bone are capillaries with direct contact with the surrounding pericytes enabling efficient communication with surrounding cells. Therefore, apart from enabling essential nutrition and oxygen diffusion and waste product removal, they are known to play a major role in bone homeostasis [15]. During bone remodelling, the positive coupling between osteogenesis and blood vessel formation is crucial as the vascular network serves as a ‘courier’ for cells delivering osteoclasts to areas where bone resorption is required to initiate bone remodelling and, at a later stage, cells from the osteoblastic lineage to promote the regeneration of bone tissue [16, 17]. Additionally, ECs can produce osteogenic factors such as bone morphogenic proteins promoting OB differentiation. Osteoclasts, OBs and osteocytes, in turn, produce pro-angiogenic factors further promoting the development of blood vessels through a positive feedback loop [16, 17]. The continuous balance between bone resorption and bone remodelling maintains homeostasis, which is crucial for healthy bone tissue.

When, due to age or bone disease, the homeostasis in bone is disrupted, the body may not be able to fully heal fractures, demanding medical treatment. For bone regeneration applications, a successful outcome depends strongly on the timely development of vasculature via angiogenesis. Over the years, a number of strategies have been explored to achieve spontaneous neovascularization in tissue-engineered grafts including controlled release of pro-angiogenic GFs, pre-seeding of biomaterials with tissue-specific cells or pre-vascularization [18–20]. Nevertheless, designing scaffolds suitable to fully guide the process of tissue regeneration, including the formation of a complex vascular network as the foundation of cell survival remains an ultimate but challenging goal and further optimization of the capillary network formation is crucial for the success of BTE. A number of review papers have considered biomaterials for BTE while others have addressed co-culture parameters to improve *in vitro* angiogenesis and vascularization strategies for BTE from a biological perspective. The focus of this review is to bring together studies of angiogenesis in an osseous environment with a particular emphasis on the selection and design of the scaffold material. In this article, we aim to clarify the state of research, focusing principally on cell behaviour and techniques relevant to the two components materials from which bone is comprised (collagen and calcium phosphates), and will describe some of contradictions observed in this complex field and identify knowledge gaps for future research.

## Biomaterials for bone tissue engineering

The choice of biomaterial has a great impact on the success of scaffolds as they direct a specific cellular response by providing distinct architectural frameworks and chemical properties altering the cell–material interactions and eventually promoting tissue regeneration. A range of different materials and composites have been studied in the field of BTE with the aim to mimic the ECM of the desired tissue. In general, five different groups of biomaterials for scaffolds can be distinguished, namely metallic, bioactive ceramics, bioactive glasses synthetic- and natural polymers and ceramic-polymer composites.

### Metal

Metal matrix scaffolds exhibit various excellent properties for bone regeneration including mechanical properties biocompatibility, thermal stability and corrosion resistance, which titanium and tantalum being most widely applied [21]. However, apart from the high cost of manufacturing, the metal implants are non-degradable, limiting their applicability for tissue regeneration. Therefore, despite the many advantages, over the years, the focus has shifted towards degradable polymer and ceramic-based materials [21].

### Bioactive ceramics and glasses

Bioactive materials have received much attention among researchers due to their capability to interact with physiological fluids, promoting the formation of bone-like HA layers, which induce a firm bond between the tissue and the biomaterial surface [22].

Bioactive glasses have been of interest in BTE due to their demonstrated capability to promote the formation of a bioactive hydroxy-carbonated apatite layer that ensures a firm bond between the bone tissue and the material surface. The exceptional surface reactivity of the material in physiological fluids is ascribed to various processes including ion leaching/exchange, dissolution of the glass network promoting precipitation and growth of a calcium-deficient carbonated apatite [22, 23]. In particular, the delivery of silicon has been associated with the activation of gene transduction pathways that stimulate cell differentiation and osteogenesis [22, 23]. Additionally, research on bioactive glass has shown its ability to promote angiogenesis. 45S5 bioactive glass particle coatings have been associated with increased VEGF production by human fibroblasts and improved EC proliferation [24]. Greater neovascularization was achieved *in vivo* after 2 weeks of implantation of VEGF-releasing biodegradable PLGA scaffolds with 45S5 bioactive glass particles and collagen sponges loaded with 45S5 bioactive glass in Lewis rats and Sprague–Dawley rats, respectively [24].

Well-known examples of bioceramics include calcium sulphates and calcium phosphates such as tricalcium phosphate (TCP) and (substituted) HA-based materials [1]. Calcium sulphate bone grafts have the fastest degradation rate and, under dry conditions, exhibit greater compressive strength and a slightly lower tensile strength than cancellous bone. However, within a moist environment, calcium sulphate tends to lose its favourable mechanical properties restricting its use *in vivo* to contained areas such as bone voids and its degradation rate is considered to be too fast for BTE [25].

Calcium phosphates, such as α-TCP, β-TCP and synthetic (substituted) HA, on the other hand, exhibit good compressive strength under both dry and wet conditions and are biocompatible, osteoinductive and osteoconductive [25]. Natural HA-based bone substitutes, such as Bio-Oss^®^ are often derived from animals and provide a highly osteoconductive environment but can exhibit a limited resorptive potential [26].

TCP is similar in composition to the main mineral component of bone but due to the fast degradation rate, resulting from its particular crystallographic structure, bone formation on these grafts is restricted [25, 27]. Synthetic HA closely mimics the natural mineral component of bone but can be considered not resorbable with a resorption rate of only 1–2% per year [25]. Additionally, Unger *et al.* [28] showed *in vitro* long-term survival of a microcapillary network grown on HA surfaces, using a co-culture of hOBs and ECs. However, phase-pure HA does lack numerous ionic substitutions found in natural bone tissue which is suggested to account for its low degradation rate and insufficient osseointegration [29]. Research has attempted to improve the bioactivity of HA by adding various ions into the crystal lattice, with popular substitutions being ${\text{SiO}}_{4}^{4-}$, Mg^2+^, ${\text{CO}}_{3}^{2-}$, Sr^2+^, Ag^+^, F^−^ and Zn^2+^. Of all substitutions ${\text{SiO}}_{4}^{4-}$ is one of the most extensively studied due to the promising results reported in the literature [30]. Silicon, long believed to be an insignificant trace element, is now known as an indispensable element of healthy bone with animal studies showing an increased incidence of skeletal defects, thinner legs and decreased mineral content in animals with silicon deprivation [31]. Similarly in humans, higher bone mineral density was associated with a higher intake of dietary silicon [31]. Overall, silicon-substituted HA (Si-HA) has been found to exhibit enhanced reactivity, bioactivity and hardness, which may result from a combination of factors influenced by the addition of ${\text{SiO}}_{4}^{4-}$ [32]. Additionally, the addition of silicon into the HA lattice has been reported to improve its bioactivity and neovascularization *in vivo* [33–35]. A study carried out in chicken embryos found Si-HA to be a good material for the conduction of blood vessels [36]. Various other studies have reported on the positive effects of silicon on angiogenesis *in vitro* using different biomaterials such as akermanite and calcium silicate [37, 38].

Another way by which the benefits of various ions could be reaped is through substitutes derived from natural bone minerals. These substitutes naturally contain various ionic substitutions and are highly osteoconductive, which can be beneficial for the bone healing process. In terms of angiogenic potential, a clinical study using the natural bone mineral Bio-Oss^®^ for sinus augmentation found that this material seemed to increase the microvessel density at the site of implantation after 6 months compared with the pre-existing subantral bone [39]. Nevertheless, while Bio-Oss^®^ does up-regulate VEGF production of certain cells and promote the formation of tubular structures, their angiogenic potential was lower compared to other xenogeneic bone grafts, namely Gen-Os^®^ of equine and porcine origins.

Apart from the many advantages of bioceramics, the production of scaffolds from these materials is often difficult and they are brittle. Some alternative materials that are easier to process include polymers.

### Synthetic and biological polymers

Numerous synthetic polymers including polylactide acid (PLA), polyglycolide acid (PGA) and poly-l-lactic acid (PLLA) have also been investigated extensively for scaffold fabrication. This is due to the tunable degradation behaviour using different variations of a polymer or a composition of different polymers, thermal plasticity, biocompatibility and the wide range of mechanical properties and architectures [40, 41]. PLLA is one of the most studied polymers for tissue engineering. When PLLA is implanted it will degrade through hydrolysis without the need for catalysts or enzymes, leaving the naturally occurring organic acid, lactic acid, as a degradation product. However, there is a major drawback associated with the usage of synthetic polymers as they are unable to facilitate cell attachment and proliferation which may ultimately result in rejection from the host tissue [42]. Another drawback of some polymers is the acidity of the degradation products which can initiate an inflammatory response within the surrounding tissue and may consequently lead to cell and tissue necrosis [42].

Usage of natural polymers can overcome a great number of the aforementioned drawbacks. Monomers, such as collagen, chitosan and glycosaminoglycans are naturally found in the ECM of many tissues. Unlike synthetic polymers, they are biologically active, containing surface peptides and ligands, and hence promote beneficial cell-scaffold interactions [43]. Moreover, they are biodegradable, have non-toxic degradation products and, most importantly, allow the host tissue to gradually replace the scaffold material with its own ECM. The disadvantages of natural polymers are their mechanical properties, that limit their usage for load-bearing applications and issues with the production of homogeneous and reproducible structures. For BTE, collagen is one of the most widely investigated natural polymers due to its abundance in human tissue and its various excellent features such as cell-recognition sites, tunable biodegradability, remarkable biocompatibility and its abundant availability. Known for its complex supramolecular structure, collagen is a key factor in maintaining the structural and biological balance and integrity within connective tissue. These excellent and unique properties have led to the development of advanced biomaterials using collagen type I and II to mimic the tissue at both biological and structural levels resulting in good integrity and reduced rejection of biomaterials once implanted [44]. Besides providing structural stability, collagen is of crucial importance as a bioactive surface through the exposure of specific cell adhesion sequences.

Overall, the abundance of collagen in human tissue and the existence of a variety of high-affinity integrin recognition sequences make it ideal for tissue engineering. Nevertheless, relatively weak mechanical properties and low thermal stability often necessitate post-processing such as chemical- and physical crosslinking, which can affect the cell response adversely as a result of a decrease in the number of available cell adhesion sites. To date, uncertainty still exists with regard to the optimal balance between mechanical stability and appropriate cell response of collagen scaffolds demanding further research.

### Composites

As bone consists of both an inorganic and organic phase, polymer/ceramic composites are considered a strong alternative to other single-phase materials. A composite may allow the tailoring of various material properties, such as mechanical performance and degradation properties, and can overcome disadvantages encountered with other biomaterials. A polymer/ceramic composite can, for example, benefit from the toughness of a polymer phase and the compressive strength of an inorganic one [23]. Additionally, the alkalinity of the ceramic can neutralize the acidity caused by the degradation products of the synthetic polymer. A promising composite for BTE is the HA-collagen composite which has been observed to induce enhanced bone tissue formation compared with HA and TCP alone, both *in vitro* and *in vivo* [23]. Furthermore, studies have shown that this material serves as an excellent delivery system for bone morphogenetic proteins, a GF involved in OBs differentiation, and provides crucial guidance for OBs through the Arg-Gly-Asp (RGD) sequence involved in specific cell-binding through integrins. Nevertheless, further optimization to more closely mimic the complex organization of bone at a nanoscale remains challenging and, while the fracture toughness, compressive and tensile strength can be tailored, the mechanical properties achieved still do not match that of bone [23].

## Vascularization of biomaterials

Although widely addressed in the literature, inducing rapid vascular ingrowth into artificial 3D structures for tissue regeneration and replacement remains a major obstacle [45–47]. To date, researchers have achieved spontaneous neovascularization in tissue-engineered scaffolds making use of the naturally occurring inflammatory response after implantation and the release of pro-angiogenic GFs resulting from the hypoxic state of the seeded cells within the implant. Nevertheless, the growth rate of the vessels is often insufficient leading to graft failure due to avascular necrosis [48]. Vascularization, essential for nutrition and oxygen diffusion as well as the removal of waste products, is of crucial importance for the biological functionality of designed scaffolds, in particular larger-sized structures. Oxygen plays a pivotal role in cell survival and as the consumption rate of oxygen by cells is faster than its delivery at diffusion distances further away from the capillary lumen than ∼200 µm, metabolically active tissue requires the distance between blood vessels and cells to be within this range [14]. Therefore, appropriate vessel ingrowth into implantable scaffolds is critical for successful regeneration and tissue growth both *in vitro* and *in vivo*.

Considering the complexity of neovascularization, various factors need to be taken into account while designing a scaffold. These include proper consideration of cell and tissue type, oxygen diffusion rate and the overall scaffold size and its integration with the host vasculature [45]. By increasing the ability of the designed scaffolds to form new blood vessels and so induce neovascularization, cell viability within the scaffold will increase, promoting further penetration toward the centre of the scaffold. However, to allow appropriate vascularization of tissue-engineered scaffolds a close resemblance of the natural process of angiogenesis is required which constitutes a complex successive cascade of very tightly regulated interactions between various GFs and different cell types.

### Angiogenesis

The generation of early blood vessels within the human body occurs from the mesoderm by differentiation of angioblasts, a type of endothelial precursor cells involved in lumenization. This process, termed vasculogenesis, involves *novo* capillary formation and occurs even before the heart starts beating during embryogenesis [49]. On the other hand, the process of angiogenesis, differentiating itself from vasculogenesis by the sprouting of capillaries from pre-existing blood vessels, occurs in direct response to tissue demands. Both processes are controlled by different molecular mechanisms, which involve distinct and precisely timed signals driven by the release of various GFs under specific conditions [49].

**Figure 3.**
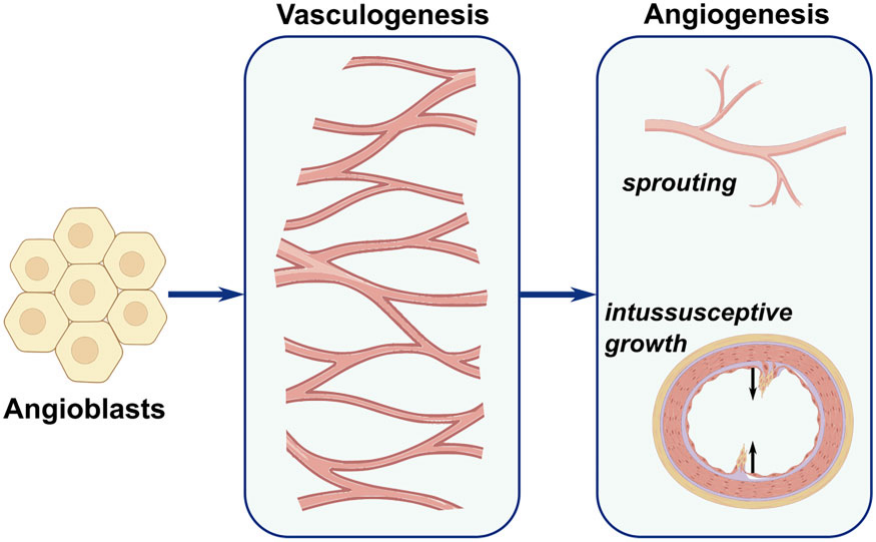
Schematic illustrating the development of neovascularization and, in particular, the morphological event of sprouting and intussusceptive angiogenesis. Sprouting angiogenesis constitutes sprouts of ECs and is responsible for the formation of new capillaries, while intussusceptive angiogenesis involves the formation of blood vessels by the mechanism of splitting. Adapted from Ref. [52].

There are two types of angiogenesis: sprouting and splitting (also known as intussusceptive angiogenesis). Sprouting is well understood and investigated compared with intussusceptive angiogenesis, which was only discovered two decades ago [50, 51]. The basic mechanism of both types is illustrated in Fig. 3. Sprouting angiogenesis, as implied by its name, constitutes sprouts of ECs, often growing towards angiogenic GFs and is responsible for the formation of new capillaries throughout life. Intussusceptive angiogenesis involves the formation of blood vessels by the mechanism of splitting and is believed to be a more efficient type of angiogenesis [51].

During sprouting angiogenesis, a cascade of several steps occurs in a specific order; enzymatic degradation of the capillary basement membrane, EC proliferation, directed migration of ECs, the formation of EC tubes, vessel fusion, vessel pruning and pericyte stabilization [53]. A cascade of precisely timed signals with a distinct concentration initiated by various inducers and factors, including soluble GFs, membrane-bound proteins, cell–matrices and cell–cell interactions, marks the start of the vascularization process [54].

Vascular endothelial GF-A (VEGF-A) also termed VEGF is one of the most extensively studied soluble GFs of the VEGF family. The biological performance relies on its interaction with cell surface receptors VEGF receptor-1 and VEGF receptor-2 expressed on vascular ECs [55]. Furthermore, VEGF-A plays a pivotal role in angiogenesis by inducing proliferation, sprouting and tube formation of ECs [56]. Other soluble GFs which appear to have a great influence on angiogenesis are acidic and basic fibroblast GFs (aFGFs and bFGFs, respectively). These are heparin-binding proteins and are known to stimulate EC proliferation and migration [57]. Moreover, membrane-bound proteins, such as α_*v*_β_3_ integrin VE-cadherin as well as platelet-derived GFs (PDGF) influence steps involved in blood vessel assembly [58]. Many other factors and proteins have been implicated to promote angiogenesis but their effect is much less profound.

### Strategies for neovascularization of scaffolds

Over the past decades, various strategies have been developed for the vascularization of 3D tissue-engineered constructs and a great deal of research has gone into understanding their mechanism and parameters. While the main focus in the literature has been on ensuring adequate tissue metabolism and sufficient oxygen exchange through scaffold prevascularization, it is also important to carefully address subsequent remodelling and adaptation of the engineered vasculature network to the desired functional requirements once implanted, which may impose new challenges. Since the angiogenic potential of many materials, including bioceramics and natural polymers, is limited, many efforts have been undertaken to improve the angiogenesis of tissue-engineered scaffolds. Strategies involve altering biochemistry and/or structural properties of the constructs or through the supply of angiogenic factors [59, 60]. Of great interest in this area of research are the GF-releasing scaffolds, cell-based strategies, and prevascularization.

#### Growth factor-releasing scaffolds

The use of various pro-angiogenic GFs to induce neovascularization, either by surface functionalization or by embedding them into a 3D construct allowing controlled release through biomaterial degradation, has been studied both *in vitro* and *in vivo*. The incorporation of pro-angiogenic GFs such as VEGF, bFGF and PDGF in scaffolds is considered important for mimicking the natural neovascularization response, as they are known to initiate and promote angiogenesis in a cascade of various steps in the ECM and hence improve neovascularization.

Through the years, various single GF-releasing systems have been examined both *in vitro* and *in vivo* with different types of GFs and scaffold materials and modes of GF loading. Sun *et al.* investigated the angiogenesis behaviour of 85:15 poly(lactide-co-glycolide) (PLG) scaffolds incorporated with VEGF implanted in the ischemic hind limb of mice. The sustained VEGF delivery of PLG-VEGF scaffolds did not only improve the tissue perfusion but also the capillary density and the maturity of the vasculature [18]. Similar results were observed with bFGF-embedded gelatin hydrogels transplanted at the same site in rabbits. After four weeks, angiogenesis improved considerably, manifested by tissue blood flow, the number of arterioles and vascular density [61]. While overall the direct incorporation of VEGF has shown promising results, the rapid initial release restricts control over the timing of GF delivery necessary to mimic the natural cascade of events.

To overcome the limitation associated with direct incorporation, a promising new method for alginate scaffolds has been developed using microspheres of poly(lactide-co-glycolide acid) (PLGA) encapsulating bFGF [62]. The method resulted in the controlled delivery of the bFGF from the alginate scaffolds which led to more extensive vascular ingrowth compared with controls [62]. Moreover, the vascular density of the scaffolds had increased after 21 days compared with 10 days justifying that prolonged angiogenesis is achievable using this method [62]. Another study conducted by Moya *et al.* [63], using aFGF-loaded alginate micro-beads in collagen scaffolds, showed not only an increase in the initial vessel formation but also showed prolonged and persistent neovascularization. Initially, in an *in vitro* study, they showed that the micro-beads in a 3D collagen gel stimulated greater microvascular network formation compared with a single bolus of aFGF [63]. A subsequent *in vivo* study in rats confirmed their findings, as aFGF loaded beads showed a significant improvement in vessel density after 6 weeks of implantation compared with bolus administration of aFGF [63]. Moreover, pre-encapsulated VEGF in PLG microspheres within PLG scaffolds has been tested against direct incorporation of VEGF [64]. Pre-encapsulated VEGF resulted in the protein being more deeply embedded into the scaffold causing a slower release kinetic as opposed to direct incorporation. Overall, a localized release of VEGF from both types of PLG scaffolds was detected with little systemic exposure [64]. The authors suggested that a combination of both incorporation techniques as well as altering polymer properties and process parameters could serve as an effective method for tailoring the release kinetics of scaffolds [64]. Overall, the localized delivery of the GFs in a controlled manner has shown significant improvement with respect to both vasculogenesis and angiogenesis.

Although promising, an alternative method involving multiple GF release has been developed as results obtained through single GF-releasing strategies demanded further optimization in terms of capillary and EC stabilization and spatio-temporal release of pro-angiogenic factors. Carmeliet and Conway [65] developed a smart system to release VEGF and platelet-derived GF (B subunits) (PDGF-BB) from a polymer scaffold. The two-phase release mechanism consists of VEGF mixed directly with the scaffold polymer for rapid release resulting in the outgrowth of endothelial channels followed by PDGF-BB release pre-encapsulated in microspheres embedded within the scaffold stabilizing the nascent vessels by utilizing smooth muscle cells [65]. An *in vivo* comparison study of a bi-layered PLG scaffold, one layer containing VEGF and the other layer a combination of VEGF and PDGF-BB, showed that the multi-GF-release strategy induced neovascularization with significantly enhanced vessel size and capillary maturity compared with a single release approach [66].

Regardless of the type of GF, spatio-temporal GF-releasing strategies to regulate the cell functionality have successfully paved the way for the improvement of vessel formation in scaffolds as it promotes cell migration, proliferation and phenotypic maturation and, ultimately, increases the scaffold survival upon implantation [67, 68]. Nevertheless, a few shortcomings concerning GF functionalization of scaffolds remain present. Although many studies confirmed the controlled release of GFs, much uncertainty still exists regarding the dose-effect ratio. Moreover, suitable factors and delivery vehicles, as well as appropriate timing, have still not been identified [69].

#### Cell-based strategies

Utilizing cells to compensate for the limitations identified for GF delivery strategies to further promote angiogenesis in tissue-engineered scaffolds has become of particular interest for vascular research. To activate the ingrowth of vessels, cells isolated from a patient’s tissues as well as genetically engineered cells have been assessed. Bajada *et al.* [19] applied a cell-based strategy to a challenging clinical case of tibial non-union. Bone marrow stromal cells (BMSCs) were harvested and isolated from a patient’s spine and cultured for 3 weeks [19]. The cultured BMSCs were combined with osteoinductive calcium sulphate (CaSO_2_) in pellet form, acting as a carrier, and implanted to fill the void in the patient’s tibia [19]. The patient was discharged 3 days post-operation and was able to place full weight on the affected leg by week 8 [19]. At this stage, no traces of CaSO_2_ were detectable and the bone had fully recovered [19]. The clinician suggested that the novel combination of BMSCs and CaSO_2_ allowed improved bone integration and vascular invasion at the site of trauma owing to the rapid degradation behaviour of CaSO_2_ [19].

A further promising cell-based approach involves the transfection of cells to overexpress angiogenic GFs. In an *in vivo* study, Ad-hVEGF165-transfected and non-transfected MSCs were implanted into the injured zone of an ischaemic rat heart [70]. A month post-implantation, greater levels of VEGF followed by significantly improved capillary network formation were detected around the injured area using transfected MSCs as opposed to non-transfected MSCs and controls, resulting in a reduction of infarct size and a stable heart function [70].

Although these cell-based strategies demonstrate good vascularization potential, particularly compared with GF-releasing approaches, there exists a limitation regarding the scale at which it can operate as a very large number of cells is required for scaffolds of clinically relevant size. More importantly, pre-culturing of scaffolds with any variety of ECs has proved to be ineffective due to their inability to proliferate and survive in a long-term mono-culture as well as their lack of self-assembly required to form microvascular networks [71].

#### Prevascularization

Neovascularization of scaffolds using angiogenic factor delivery or cell-based strategies may take up to several weeks and relies entirely on ingrowth of the host tissue for the formation of capillaries. An alternative strategy, termed prevascularization, has the advantage of having pre-formed capillaries within the 3D construct prior to implantation and, thus, a shorter time is needed to fully adapt to the body. The pre-implantation period can take place *in vitro* as well as *in vivo*.

**Figure 4.**
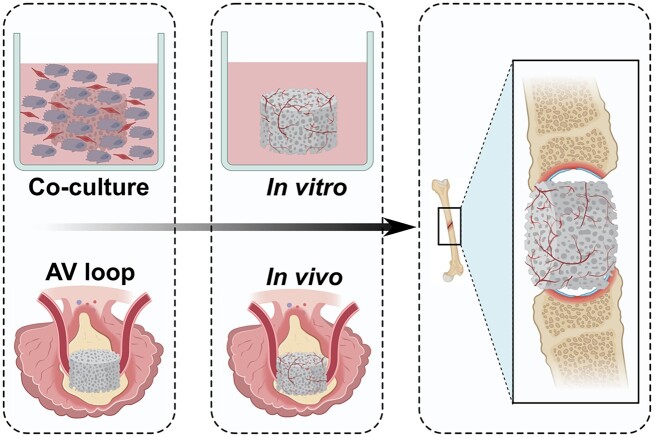
Schematic comparison of an *in vitro* endothelial co-culture versus an *in vivo* AV loop method for prevascularization of scaffolds for BTE.

*In vivo* prevascularization consists of two distinct steps. The first step involves the implantation of a scaffold adjacent to an artery for blood supply. The surrounding artery will supply all the necessary nutrients for the capillaries to grow naturally and so several weeks are required for the formation of the microvessel network [72]. In the next stage, the implanted scaffold including the supplying artery is harvested and re-implanted into the target site, with the vascular axis being connected to local vasculature through microvascular anastomosis, resulting in immediate perfusion of the scaffold [73]. Dvir *et al.* [20] seeded alginate scaffolds with neonatal cardiac cells and a mixture of pro-angiogenic GFs including VEGF and cultured them for 48 h to allow initial tissue organization prior to transplantation into rat omentum. The cardiac patches were harvested after 7 days. The prevascularized patches were subsequently transplanted into infarcted rat hearts. After 28 days of implantation, the preformed vasculature network was able to both structurally and electrically integrate with the host myocardium [20]. Similarly, bladder-shaped collagen and collagen-PGA composite scaffolds were seeded with biopsied urothelial and muscle cells for 7 weeks before being implanted with- or without an omental wrap [74]. The greatest bladder function improvement was achieved amongst patients receiving an omental wrap containing scaffold, as it resulted in increased ingrowth of newly formed vessels by supporting and maintaining the transplanted cells [74]. An alternative source for prevascularizing scaffolds *in vivo* is the usage of the arteriovenous (AV) loop. Hereby, a loop is constructed between an artery and a vein by interposing an autologous vein or a synthetic equivalent which can subsequently be wrapped around the scaffold as illustrated in Fig. 4. Tanaka *et al.* [75] successfully prevascularized an artificial skin dermis in rats using an AV loop. Active formation of a newly formed vasculature network originating from the loop vessel resulted in the formation of new tissue after 4 weeks [75]. Although *in vivo* prevascularization seems to be a promising method, the drawbacks, such as the need for multiple surgeries as well as the potential need for removal of a vascular axis from the initial implantation site should not be overlooked when considering clinical applications.

*In vitro* prevascularization strategies appear to be a promising alternative. Essentially, two major strategies exist for *in vitro* vascular network formation. While the first method uses isolated intact microvessels, which were found to retain their angiogenic potential, the second one, illustrated in Fig. 4, uses a co-culture of ECs and supporting cells to allow for the initial formation of an entirely new microvascular network. Upon implantation, both pre-formed networks allow outgrowth of blood vessels from the inner region of the scaffolds, ultimately resulting in anastomosis with the host vasculature. In a study conducted by Shepherd *et al.* [76], microvessel fragments of a male rat were isolated and cultured in collagen I gel for 5–7 days to allow initial microvessel network formation prior to implantation in female mice. Initial angiogenic sprouting at the end- and mid-regions of individual fragments in the collagen gel was observed *in vitro*. Vessel assembly started post-implantation within the first 3 days culminating in a mature-appearing vascular bed after 28 days of implantation [76]. In a more recently conducted study, a comparison was made between single-cell stromal vascular fraction (SVF) of adipose tissue and adipose tissue-derived microvascular fragments (ad-MVFs) representing a mixture of intact arteriolar, capillary and venular vessel fractions [77]. It was observed that *in vitro* prevascularization of collagen-glycosaminoglycan matrices seeded with ad-MVF resulted in a significantly higher functional microvessel density in mice after 2 weeks of implantation compared with SVF-seeded scaffolds [77]. The authors suggested that, amongst other reasons, ad-MVF accelerated vascular formation in scaffolds due to the presence of fully functional vessel segments [77]. Although widely regarded as a promising vascularization strategy, a study by Laschke *et al.* suggested restricted usage in aged patients. Results obtained in this study revealed that adipose-derived fragments obtained from aged mice donors (16 months) seeded onto 3D constructs showed a significantly reduced functionality resulting in a reduced microvessel density and intravascular blood flow velocity as opposed to fragments obtained from adult mice donors (8 months). The authors attributed this mainly to the observed lower number of matrix metalloproteinase-9-positive perivascular cells in fragments from aged donors as opposed to adult donors as no significant differences could be found in stem cell content and proliferative activity as well as EC survival and the release of angiogenic GFs under hypoxic conditions [78]. Other studies did observe a negative effect of ageing on the proliferative activity of stem cells and so further research should be carried out to measure the extent of this effect on fragment functionality [78].

Although using isolated intact microvessels circumvents the need for *novo* vessel formation, only a few experimental studies have analysed their regenerative potential since 1998 and much remains unknown [79]. On the other hand, a great deal of research has focussed on *in vitro* strategies using stem cells in co-culture. Tremblay *et al.* [80] demonstrated capillary formation in collagen sponges for skin applications cultured with human keratinocytes, human dermal fibroblasts and human umbilical vein ECs (HUVECs). A more important observation was the improved perfusion in the prevascularized grafts detected after just 4 days of implantation in mice as opposed to non-prevascularized scaffolds which reached the same status after 14 days post-implantation [80]. Moreover, *in vitro* prevascularized polymer constructs seeded with a 3D multi-culture system containing myoblasts, embryonic fibroblasts and ECs demonstrated anastomosis and were found to enhance perfusion and cell survival [81]. In a more recent study, Mishra *et al.* [82] evaluated the effect of *in vitro* prevascularization on *in vivo* vascularization. Poly(propylene fumarate)/fibrin composite scaffolds were co-cultured with MSCs and HUVECs prior to implantation in an immunodeficient mouse model using 5 different conditions: no pre-culture, 1-week pre-culture, 2-week preculture, 3-week pre-culture and scaffolds without cells serving as a control [82]. The authors observed a significant increase in cellular outgrowth and vascular network density *in vitro* with increasing pre-culture time and after a 2-week pre-culture formation of lumen-like structures could be distinguished [82]. Increased pre-culture time was also found to improve the *in vivo* vascularization capacity and lumen formation considerably, emphasizing the importance of prevascularization using a co-culture system [82].

Considering all the evidence available, it appears that *in vitro* pre-culturing of tissue-engineered constructs for an initial assembly of a microvascular network, using either a mixture of microvessel fractions or cells in co-culture, significantly improves ingrowth, density and maturity of vessels formed post-implantation. Overall, a co-culture of ECs with supporting cells provides a solid and reliable *in vitro* strategy to establish microvascular-like structures, avoiding the need for multiple surgeries, and can, more fundamentally, serve as a strong *in vitro* model to study cell–cell and cell–material interactions.

#### Endothelial co-culture

For many years, mono-culture studies have been of key interest in the field of tissue engineering providing physicochemical evidence regarding cell growth and cell–material interaction. However, attempts to promote stable angiogenesis by mono-culturing ECs on scaffolds have failed due to the inability of ECs to survive, proliferate and self-assemble into capillary-like structures when in mono-culture [83]. This resulted in a shift of focus towards coculture approaches more closely resembling natural tissue both physically and biologically without losing the benefits of a mono-culture. A co-culture system, with a given degree of contact between different cell populations, enables natural intercellular interactions and mimics both the organization and complexity of *in vivo* environments and increases the culturing success for certain cell populations, e.g. ECs [84]. The main advantage of co-culture systems is their ability to assess the regulatory mechanisms at the level of cellular cross-talk which is mainly mediated by secreted factors, cell–cell interactions and cell–ECM communication [84]. Moving forward, researchers have attempted to further improve the resemblance of the system to natural tissue by introducing multi-culture systems increasing the level of complexity in terms of molecular interaction but, to date, such systems remain unpredictable and difficult to manage [85]. Overall, a co-culture of two cell types has been used in the majority of the studies.

Various types of ECs are involved in the circulatory system and hence are of key importance for angiogenesis and vascularization of tissue-engineered grafts. Therefore, co-cultures consisting of ECs with supporting parenchymal cells are of great interest for vasculature formation in scaffolds, but obtaining the right balance between the many interlinked variables within the system such as cell type, culture medium, and cell ratio for successful *in vitro* co-culturing remains a complex process.

#### Cell type and source

Close consideration of cell type and cell source is essential for establishing a functional co-culture system. Variables significantly affecting this complex system include cell origin, differentiation stage and cell culture type. Cell types include primary cells obtained directly from human or animal tissue and cell lines which are continually passaged resulting in acquired homogenous genotypic and phenotypic characteristics. The choice of a particular cell type is pivotal but not always straightforward. Within a cell group, for example, ECs, a variety of specialized differentiated types may occur which can fundamentally change the outcome of a co-culture system. Care should be taken to ensure that the cell type meets the specific requirements of the targeted microenvironment. Previous research observed that although tubulogenesis was detected for all types of ECs, they all possess a distinct tissue-specific expression profile depending on their origin [86]. The difference in gene expression is believed to be the driving force for specific vascular growth, resulting in capillary formation in the case of human dermal microvascular ECs (HDMECs) and larger vessel formation for HUVECs. Apart from the expression profile, the differentiation state of ECs also has a great impact on vascular network formation. Previous research using endothelial progenitor cells (EPCs) in co-culture with human MSCs showed that prevascular structures were formed only when endothelial differentiation was at a mature state [87].

A further important aspect of establishing a functional co-culture system is the choice of supporting cells as they can substantially influence capillary formation. In the case of ECs, no vascular network formation can be obtained in mono-culture without the addition of supplements, while the formation of vessel-like structures for ECs in co-culture with fibroblast or OB lineages with and without supplements has been established widely [88, 89]. The supporting cells are known to express angiogenic factors and hence promote the formation of capillary-like structures. Santos *et al.* [90] showed that the expression of VEGF observed in a primary human OB (hOB) co-culture with HDMECs was higher than detected in the hOB mono-culture. This is believed to be due to a paracrine mechanism of VEGF-stimulated ECs which promotes the VEGF release in OBs as a result of direct cell contact through the gap junction protein connexin 43 [90].

#### Cell ratio

In a co-culture, the cell ratio has a great impact on cell–cell and cell–matrix interactions and is the most crucial experimental variable determining the success of the system. However, no optimal cell ratio has yet been reported for ECs co-cultured with OBs or fibroblasts and no material-specific consensus can be extracted from literature as seen in Table 1. Since the ‘optimal’ cell ratio is dependent on the desired outcome for a particular application this variable cannot easily be generalized. For example, if the expression of a specific phenotype, such as an osteogenic marker, is desired, an increased proportion of the supporting cells, in this case, hOBs, would be favourable, while poor performance of ECs on particular biomaterials would demand an increased proportion of ECs [28, 91, 92].

**Table 1.** A summary of different culturing variables applied to co-culture systems, targeting vascularization of scaffolds for BTE

Cell types		Cell ratio	Culture medium	Lumen formation	Seeding logistic	Scaffold material	Refs
HUVEC	hOB	1:1	PM	Yes	Simul.	Polyurethane	[89]
EPC	MSC	1:1	PM	Yes	Simul.	FN-c. β-TCP	[96]
HUVEC	MSC	2:1	PM	No	Simul.	Well plate	[97]
HUVEC	hOB	1:5	PM	No	Simul.	Well plate	[98]
HUVEC	hOP	2:1	PM	Yes	Simul.	Alginate	[99]
EPC	MSC	1:1	PM	Yes	Simul.	Matrigel	[100]
HUVEC	MSC	3:1	PM	No	Seq.	Well plate	[101]
HUVEC	MSC	1:5	OM	No	Simul.	Well plate	[102]
HUVEC	MSC	2:98	OM	No	Simul.	Well plate	[103]
EPC	MSC	1:1	OM	Yes	Simul.	PCL-TCP	[104]
HDMEC	MSC	1:1	OM	Yes	Simul.	Cu^2+^-doped BG	[105]
EPC	MSC	1:2	OM	N/a	Seq.	PCL-HA	[106]
HDMEC	hOB	13:2	EM	Yes	Simul.	Silk fibroin	[91]
HDMEC	hOB	17:3	EM	Yes	Simul.	HA, NiTi, TCP	[28]
HDMEC	hOB	5:1	EM	Yes	Simul.	Collagen	[107]
HDMEC	hOB	4:1	EM	Yes	Simul.	Starch PCL	[90]
OEC	hOB	1:1	EM	Yes	Simul.	Starch PCL	[108]
OEC	hOB	1:1	EM	Yes	Seq.	Silk fibroin	[109]
OEC	hOB	2:3	EM	N/A	Seq.	FN-c. CS	[110]
OEC	hOB	1:1	EM	Yes	Seq.	Collagen	[111]
HDMEC	hOB	1:1	EM+OM	Yes	Simul.	Agarose	[112]
HDMEC	hOB	1:1	EM+OM	Yes	Simul.	Agarose	[113]
HDMEC	hOB	4:1	EM+OM	Yes	Simul.	Agarose	[114]

EM, endothelial cell medium; EPC, endothelial progenitor cell; HDMEC, human dermal microvascular endothelial cell; hOB, primary human osteoblast; hOP, human osteoprogenitors; HUVEC, human umbilical vein endothelial cell; MSC, mesenchymal stromal/stem cell; OEC, outgrowth endothelial cell; OM, osteogenic medium; PM, proliferation medium; seq., sequential; simul., simultaneous.

In general, the optimal cell ratio for a co-culture system depends on characteristics such as metabolic activity and proliferation behaviour of cells as well as the material type and surface structure. A close consideration of these features may prevent one cell type from overpopulating or even starving the less metabolically active type. Monoculture studies provide a simple tool to obtain initial insight into proliferation and metabolic activity of the cell types, however, the behaviour of these cells in co-culture is considerably different and, thus, cell-specific characterization directly within such a complex system is necessary [93]. Although a major challenge is to quantify the contribution of each cell type, cell-specific detaching methods, such as magnetic sorting or fluorescent activated cell sorting, could serve as effective ways to distinguish cells in co-culture.

#### Culture medium

Culture medium is another significant aspect of *in vitro* cultivation as it provides nutrients, oxygen and other chemical supplements necessary for cell survival and directs the cell expression profile. Generally, to maintain a particular phenotype and cell morphology, the addition of supplements is required. In literature, depending on the source and the differentiation state of ECs, various supplements have been successfully implemented in mono-culture including ascorbic acid, hydrocortisone, stromal cell-derived factor-1, epidermal GF (EGF) and VEGF [93]. However, since the expression profile of cells in co-culture changes, the effects of these supplements can become unpredictable and/or undesirable. This is exemplified in the work of Unger *et al.* [28], where the addition of angiogenic factors, such as bFGF or VEGF, in a mono-culture of HDMECs, resulted in the formation of capillary-like structures, while these supplements failed to induce microcapillary formation in a co-culture of HDMECs and hOBs. Consequently, the choice of adequate supplements for cells in co-culture is complex and requires thorough consideration.

An additional significant challenge regarding culture media for co-culturing involves establishing an optimal compromise between cell-type-specific media to serve each cell type with adequate and necessary components so that its phenotype will be kept. Various approaches exist to successfully accommodate both cell types, including the mixing of cell type-specific media according to the respective cell ratio or choosing a slightly adjusted version of the culture media used for the more sensitive cell type, by adding supplements to satisfy the needs of the other cell population. As seen in Table 1, various types of cell culture media have been investigated. In general, studies investigating the vascularization behaviour of ECs co-cultured with hOBs, focussing on angiogenic interaction rather than mineralization, primarily use the media of the more sensitive ECs [90, 94].

#### Seeding logistics

The seeding strategy used to establish a co-culture, including the seeding method and cell density, has a profound influence not only on the survival odds of the grafts but also on the ability to achieve a physiological equilibrium between the different cell types. The seeding method applied to a 3D scaffold for co-culture purposes can be modulated temporally (simultaneously or sequentially) and spatially (seeding on one or more constructs), depending on the desired outcome. A spatial seeding technique, using more constructs, is favourable when the different cell phenotypes required for the co-culture demand competing scaffold characteristics. However, a major disadvantage of the method is the lack of heterotypic cell–cell contact [93]. While seeding the cells at the same time is beneficial if the cell–cell interaction is required immediately to achieve the appropriate cell function or when the cells are naturally co-located within the tissue of interest, introducing a lag between seeding of the different cell types might be needed if the proliferation rate of the cells differs significantly or if it is preferable to direct the scaffold’s characteristics towards a particular phenotype [93].

#### Co-culture state of the art

Taken together, a balance and synergy between the aforementioned factors is necessary to establish the desired physiological equilibrium and will, ultimately, determine the success of a complex co-culture system. Table 1 lists several co-culture systems and illustrates clearly how the reported optimal settings vary amongst different studies depending on the differences in the desired outcome, experimental set-up and material properties. Noteworthy is the high number of simultaneous seeding techniques, which are predominantly chosen due to the time efficiency or simply for the sake of consistency with previous approaches. Nevertheless, Iyer *et al.* [95] showed that sequential seeding strategies, allowing initial unimpeded tubulogenesis of ECs stabilized by the subsequent addition of supporting cells, can result in improved capillary tube formation. On the other hand, another study found that a pre-formed basement layer of supporting cells was found to be beneficial for the guidance of EC vessel formation towards more native-like structures [71].

**Figure 5.**
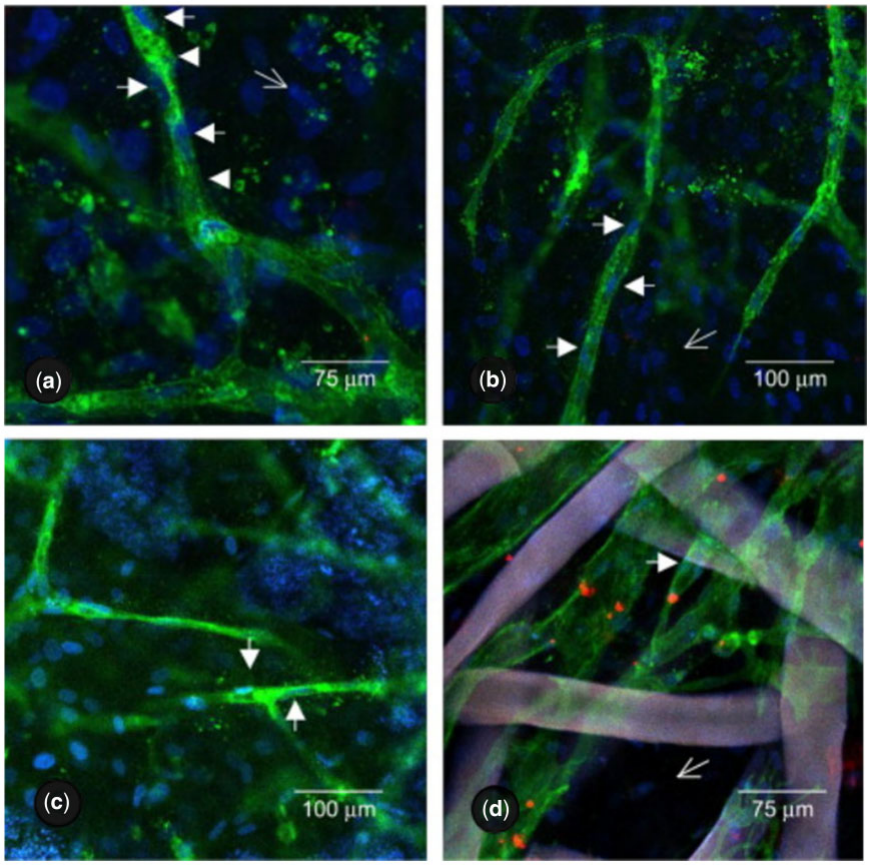
Confocal images of HDMECs in a co-culture with hOBs on porous HA (**a**), TCP (**b**), NiTi (**c**) and fibroin nets (**d**) after 42 days of culture.

A substantial contribution to co-culture research has been made by Unger *et al.*, particularly regarding co-cultures of HDMECs and hOBs. The authors investigated both cell ratio and culture media in an effort to obtain tailored approaches so as to meet the various requirements of HDMECs co-cultured with MG-63, the OB-like human cell line, or hOBs on various biomaterials, as seen in Fig. 5 [28]. They demonstrated that without the addition of exogenous angiogenic factors HDMECs were present up to at least 42 days of culturing on all materials forming microcapillary structures containing a lumen while, surprisingly, co-cultures with exogenous angiogenic factors failed to form such a network [28]. Although the authors did not offer an explanation for this phenomenon, Baldwin *et al.* [93] suggested that the addition of VEGF may have over-stimulated ECs, destabilizing the vessel formation. Observed up-regulation of GF production by supporting cells is believed to have contributed to the exceptionally high survival time [28].

Cells and nuclei are labelled with EC-specific PECAM-1 (green) and DAPI (blue), respectively. White arrowheads show PECAM1-stained capillary-like structures, including HDMEC nuclei, open arrows show the hOB nuclei. Adapted and reproduced from Ref. [28]. Copyright ©2007, Elsevier Ltd.

Subsequent research carried out by Santos *et al.* [90], co-culturing HDMECs and hOBs with a ratio of 4:1 on a blend of corn starch with poly(-caprolactone), evaluated microvessel network formation in the early and late stages of co-culturing and assessed the cross-talk between the participating cell populations. While at the early time points only monolayer patches of HDMECs were detected segregated from the OBs, microcapillary-like structures were detected after 21 days of culturing and by Day 35 a higher level of complexity was observed due to branching of the capillaries [90]. Moreover, the measured secretion of VEGF for co-cultured OBs was found to be around two to four times higher than for mono-cultured OBs. As no VEGF secretion was observed for HDMECs in monoculture this increase was suggested to be the result of heterotypic communication between the two cell types [90]. Evidently, many efforts have been made to establish various co-culture systems for clinical applications and *in vitro* modelling with the purpose of enhancing the self-assembly of vascular networks in tissue-engineered constructs [115]. Nevertheless, a golden standard for co-culture parameters does not exist as the optimal settings strongly depend on various factors including the type of biomaterial and the scaffold design.

## Designing scaffolds for tissue regeneration

**Figure 6.**
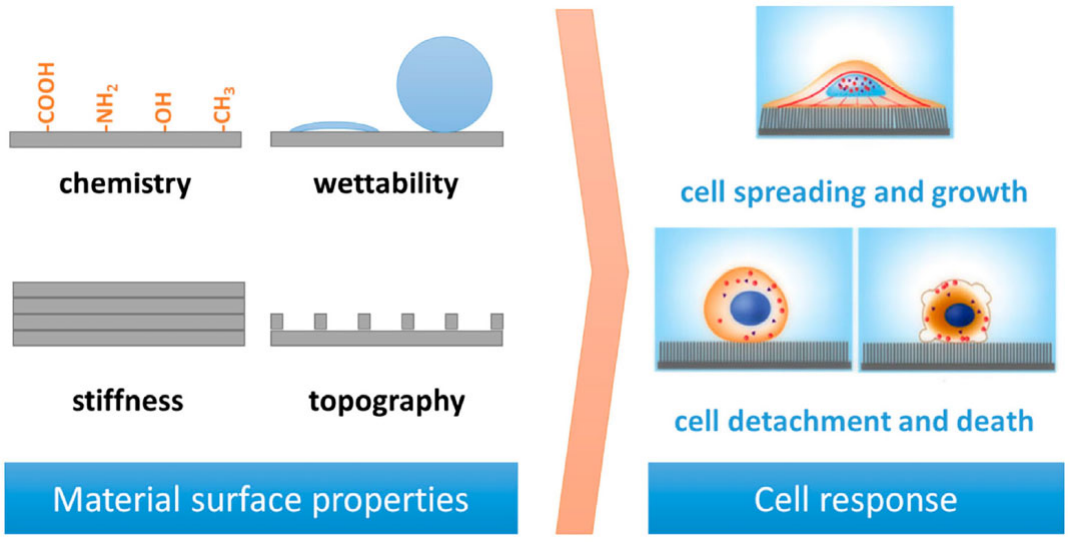
Schematic representation of the cell response to various surface features of a biomaterial, obtained from Ref. [120].

For the growth of tissue and functioning vascular networks into 3D constructs, it is not only important to carefully choose the type of biomaterial but also to fine-tune various scaffold features at the nano-, micro- and macro scale. Multiple physical and electrochemical surface characteristics such as chemistry, wettability, stiffness and topography (Fig. 6) and structural features such as interconnectivity, pore morphology and orientation have been shown to greatly influence cell adhesion, infiltration and differentiation [116–119]. Unravelling the mechanisms behind these interactions have been a focal point in BTE to enhance scaffold integration and vascular infiltration.

Optimal surface properties depend strongly on the desired outcome and the biomaterial used, but it is known that cells require a level of surface roughness to allow secure attachment and that mechanical properties should mimic the native tissue to a certain extent. Often small changes in the chemical composition or physical properties can have a significant effect on various electrochemical properties of the materials, including surface wettability and surface potential, which subsequently alters the biological response in *in vitro* or *in vivo* environments. Furthermore, sufficient porosity is necessary to initially accommodate cells in the scaffolds and ultimately promote their attachment, proliferation and differentiation resulting in enhanced tissue ingrowth [121]. Also, high (and homogeneous) pore interconnectivity in a 3D scaffold is crucial for uniform cell seeding and distribution as well as diffusion exchange of nutrients and metabolites necessary for the survival of the scaffolds *in vivo* [122]. Homogeneous and adequate interconnection of pores will result in vascularization of the bulk of the scaffolds reducing both core degradation and size limitation of produced 3D constructs [123]. Furthermore, adequate mechanical stability, a degradation rate resembling that of the hard- or soft human tissue environment (with elastic moduli in the range 0.001–20 GPa and 0.001–1 MPa, respectively) are desired to ensure a suitable environment for cells to attach and form their own ECM and to reduce the deformation or failure of the scaffolds [124]. Lastly, pore size has been shown to be crucial for the regeneration of tissue as the surface area provided in a scaffold determines the ligand density, a chemical group guiding the interaction between cells and the surface of the scaffolds. A sufficient surface area is required to allow appropriate cell migration and efficient binding of a critical number of cells to the scaffolds [118, 125]. In addition, pore size not only determines the migration of the cells into the scaffolds but also ensures the transport of cellular nutrition and waste products.

### Controlling surface properties

Over the years, surface characteristics of collagen and calcium phosphate-based biomaterials have been thoroughly assessed and compared. Small differences in the production protocol of the biomaterial may influence multiple surface properties, each of which is highly relevant for their final *in vivo* performance. Due to the intertwined nature of the various characteristics, multiple (sometimes competing) processes are influenced simultaneously requiring a thorough understanding of the relationship between various properties in order to enable the production of scaffolds with the desired characteristics.

#### Mechanical properties

Mechanical properties are of crucial importance for tissue engineering as they can direct cell behaviour such as attachment and differentiation. The specific mechanical requirements for scaffolds depend strongly on the target tissue with rigid tissue often demanding stiffer scaffolds. The mechanical characteristics of collagen can vary depending on the source, pre-treatment and processing method. Although collagen is one of the most promising materials for tissue regeneration, for some applications, such as bone replacement, collagen scaffolds, with reported Young’s moduli under tension and wet conditions ranging from 92 to 390 kPa, do not exhibit the desired structural stability and stiffness and require a stabilization process [126, 127]. An established processing step, known as cross-linking, is commonly used to overcome the mechanical instability. In natural tissue *in vivo*, collagen fibres are cross-linked enzymatically to reach the required stiffness. During the extraction process of collagen for the purpose of tissue engineering, these cross-links are disrupted leaving the collagen weak and susceptible to degradation. The strength can be partially restored by chemical or physical treatment of collagen scaffolds.

Ultraviolet (UV) radiation and dehydrothermal treatment (DHT) are the most commonly used physical crosslinking methods. DHT requires the collagen sponges to be vacuum heat treated up to 98°C for a duration of several days. The UV technique is much faster with the disadvantage that radiation penetration is limited [128, 129]. Both methods seem to cause partial fragmentation of the collagen α-chain during treatment which can subsequently affect the cell behaviour of the scaffolds [130]. Of the available chemical agents, glutaraldehyde (GTA) and 1-ethyl-3(3-dimethylaminopropyl)-carbodiimide hydrochloride (EDC) cross-linking in the presence of N-hydroxysuccinimide (NHS) are the most commonly used and investigated methods. While EDC forms so-called ‘zero-length’ cross-links, GTA cross-linking can be established between molecules that are not directly adjacent due to the long polymer chain [131]. Although it has been shown that GTA and EDC cross-linked scaffolds both have a greater compressive modulus than those stabilized via DHT, GTA is linked to the presence of cytotoxic residues [132, 133].

The mechanism of EDC cross-linking initiates activation of carboxylic acid groups of collagen via EDC resulting in the formation of O-acylisourea groups [134]. The EDC group will subsequently be replaced by NHS, forming a stable NHS-ester allowing a nucleophilic reaction of the activated carboxylic groups with primary amines on adjacent collagen molecules, hence forming ‘zero-length’ cross-links, releasing only urea derivatives as by-product [134]. Neither EDC nor NHS is incorporated in the final product and so are washed out.

**Figure 7.**
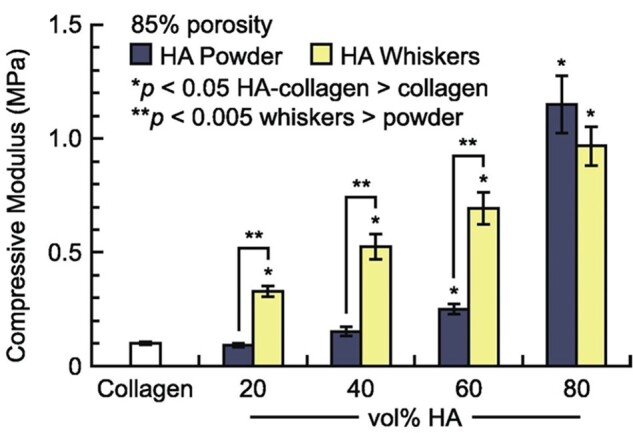
Compressive modulus of HA-Collagen scaffolds with increasing vol% HA powder or whiskers. The compressive modulus increases from 100 kPa for pure collagen scaffolds up to ∼1150 and 1000 kPa for scaffolds reinforced with 80 vol% HA powder and whiskers, respectively. Up to 60 vol% HA, whiskers have a stronger influence on the compressive strength than HA powder [135]. Copyright © 2015 Acta Materialia Inc. Published by Elsevier Ltd.

Another way by which the mechanical stability of collagen can be increased is by varying the composition of the suspensions and HA-Collagen composite scaffolds are commonly assessed for BTE. One study showed that incorporation of HA into the collagen suspension improves the Young’s modulus of the final scaffolds from 92 to 209 kPa, when measured under tensile loading conditions [127]. Depending on the wt% of HA added, the tensile strength can increase significantly with literature reporting clinically relevant Young’s moduli between 0.1 and 89 500 kPa. The compressive strength of HA-Collagen scaffolds with 85% porosity was observed to increase from 100 kPa for pure collagen to ∼1000 kPa for collagen scaffolds reinforced with 80 vol% HA (Fig. 7) [135]. While 80 vol% HA results in the highest measured compressive strength, the scaffold was found to be very brittle, making the addition of 60 vol% HA more clinically relevant as its elasticity allows full recovery of the scaffold after at least 100 000 deformation cycles of up to 50% compressive strain [135]. As seen in Fig. 7, up to 60 vol% HA, the addition of HA whiskers had a stronger influence on the compressive strength than the addition of HA powder, making this a promising option to strengthen collagen scaffolds whilst keeping their important elastic properties [135].

Pure calcium phosphates provide stiffer scaffolds but are brittle and require shaping and processing to achieve the desired mechanical properties [136]. To achieve this, various powder characteristics, including particle size, morphology and agglomerations, as well as processing parameters, such as sintering holding time and temperature can be adjusted and optimized.

**Figure 8.**
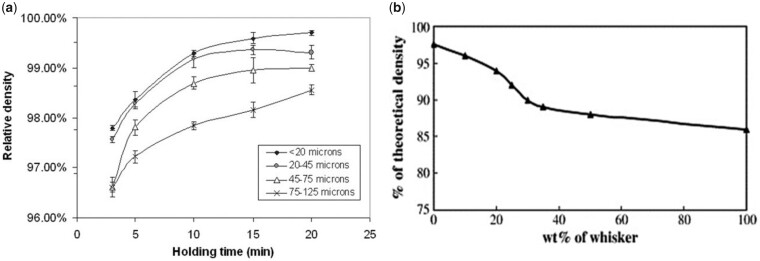
Effect of particle size and shape on disc densification. (**a**) Effect of particle size on the relative density of compacts obtained through spark plasma sintering using various holding times. Obtained from Ref. [143]. Copyright ©2007, Wiley Periodicals, Inc. (**b**) Densification behaviour of HA compacts at 1250°C using conventional sintering, with different rod-shaped (whisker) particle content. Obtained from Ref. [140]. Copyright ©2006, Elsevier B.V.

It is generally understood that smaller particle sizes provide a greater driving force for densification and this, combined with a smaller grain size after sintering, results in enhanced in a final product with enhanced mechanical properties. This trend was visible in powder compacts produced by Li *et al.* using a sintering method called spark plasma sintering (Fig. 8a) but it was less obvious in other studies using conventional sintering [137–139]. While the latter studies did recognize the general influence of smaller particles on sinterability, they also stressed the importance of other characteristics, such as crystallinity and particle size distribution and morphology on the densification efficiency of calcium phosphate powders [138]. In terms of morphology, the final packing density depends entirely on the surface energy (SE) per unit volume. As shown by Banerjee *et al.* [140] increasing the rod-shaped particle content within a compact comprising spherical nano-sized powder particles decreased the relative density of the HA compacts significantly, as rod-shaped particles have a lower SE per unit volume (Fig. 8b). An increasing number of rod-shaped particles, therefore, reduces the SE, which is the driving force for densification [140].

**Figure 9.**
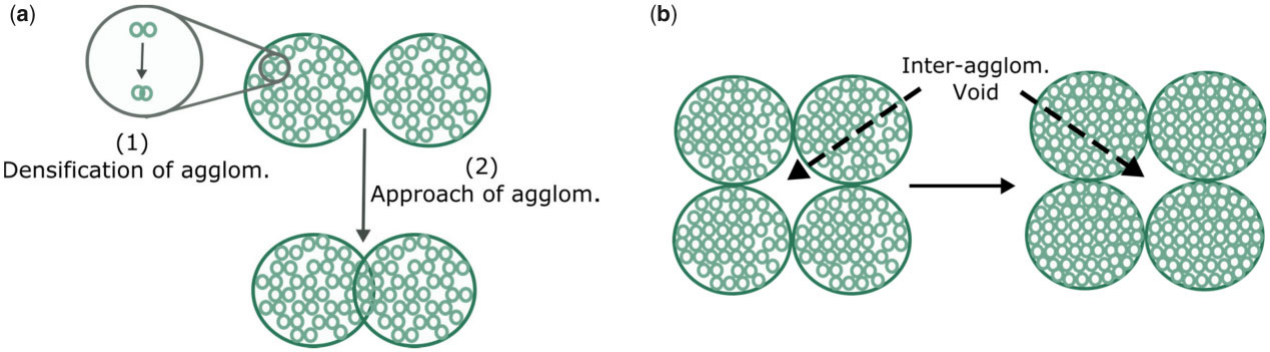
Schematic drawing showing sintering of an agglomerated ceramic powder compact with (**a**) initial stage and (**b**) intermediate and final stage sintering. Adapted and reproduced from Ref. [141].

Furthermore, the presence of agglomerates in the powders also significantly influences the packing density. During densification, the initial stage is dominated by densification of the individual agglomerates (Fig. 9a) [141]. When dense agglomerates are formed, the intermediate and final stages of densification eliminate the small remaining voids within the agglomerates and the large inter-agglomerate voids (Fig. 9b) [141]. Due to initial densification within the agglomerates, areas with much higher density compared to the surrounding matrix are formed. This reduces the interconnectivity of the pores within and between the agglomerates necessary for the elimination of remaining gaseous species, restricting further densification. Well-dispersed powders are not limited by these isolated pores and inter-agglomerate voids, resulting in higher densification, especially during the intermediate and final stages [141].

**Figure 10.**
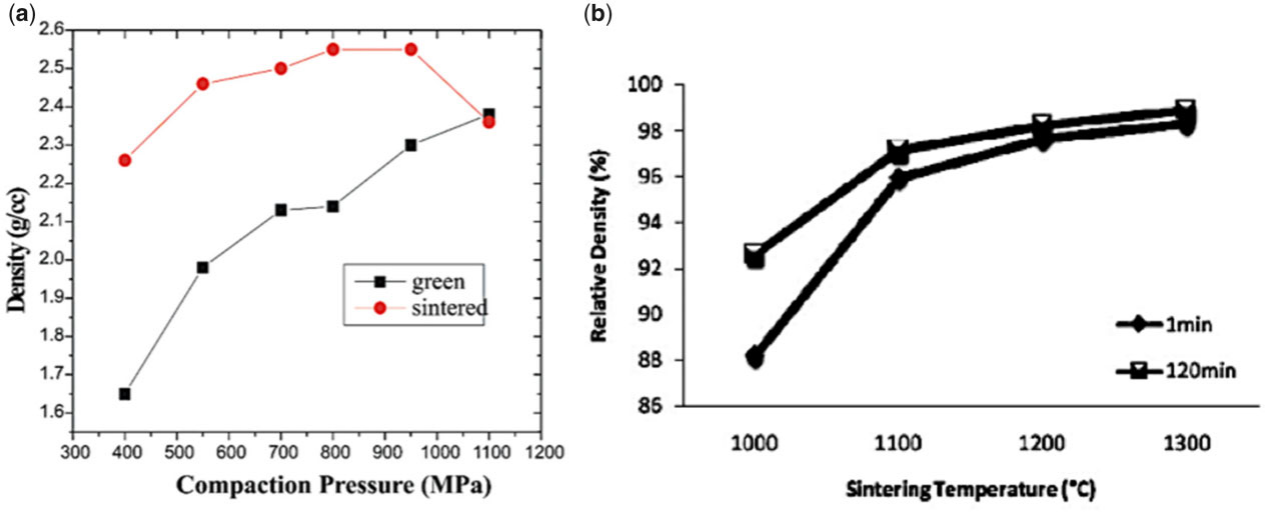
Effect of conventional sintering processing parameters on densification. (**a**) Green and sintered density of HA samples sintered at 1100°C at various compaction pressures showing a considerable increase in sintered density up to a compaction pressure of 700 MPa. Obtained from Ref. [144]. Copyright ©2005, Society for Biomaterials and Artificial Organs—India. (**b**) Relative density vs sintering temperature, showing an increase in relative density with increasing sintering temperature, and improved disc density with increasing holding time at 1000°C. Obtained from Ref. [142]. Copyright ©2011, the Automotive Engineering Centre (AEC), Universiti Malaysia Pahang.

In terms of the final sintering temperature, Fig. 10b shows that the relative density of the HA compacts increases with increasing sintering temperature, with 1300°C resulting in HA compacts at near full density [142]. The exact temperature for the onset of sintering depends on the powder type, with silicon-substituted HA (Si-HA), for example, requiring a higher sintering temperature to induce densification. Gibson *et al.* [143] observed this effect showing a significantly lower density for Si-HA discs compared with HA at temperatures between 1000 and 1150°C. However, when the densification reached a maximum (at temperatures as high as 1250°C), no significant difference in disc density was observed [143]. It remains unclear how silicon affects the sinterability of the compacts, but the authors discussed various plausible hypotheses. Firstly, as-precipitated Si-HA powder is associated with increased carbon content, and, although carbon is fully eliminated upon heat treatment, they suggested that the increased decarbonation may have affected the sintering process [143]. Secondly, it was also proposed to be associated with the vacancies at the hydroxyl sites, as a result of the silicon substitution. According to the authors, it may be the case that the remaining hydroxyl groups associated more strongly with the calcium and silicate groups in Si-HA as opposed to HA, which prevented densification due to reduced mobility of these groups. When a certain temperature was achieved, the mobility may have increased enough that the sintering process began to match that of HA [143].

#### Grain size

**Figure 11.**
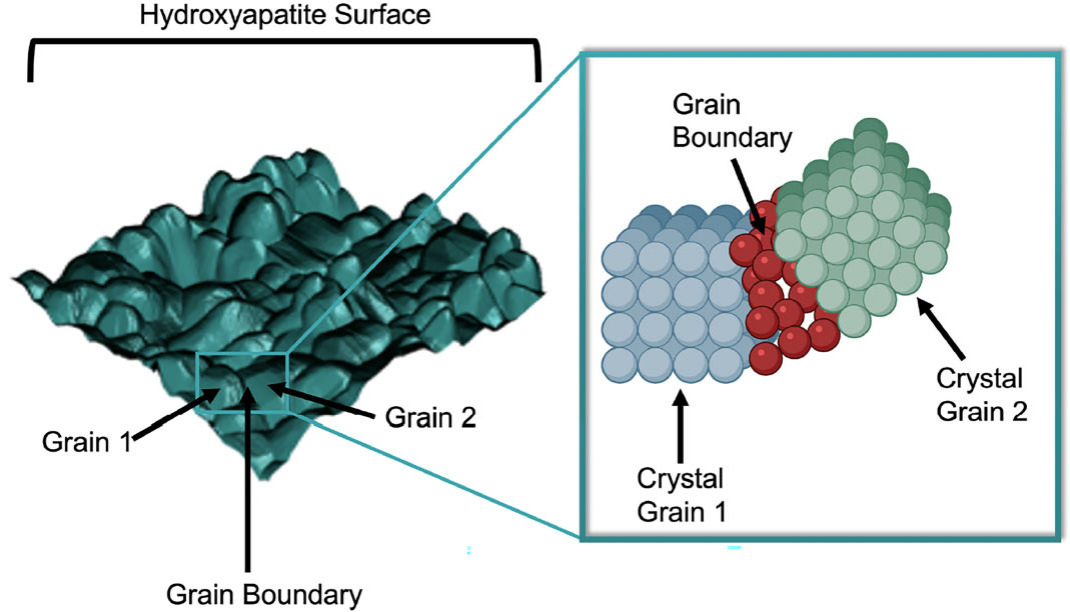
Schematic of crystalline grains and amorphous grain boundaries on a surface and their respective lattice structure.

Grain size is another relevant characteristic influencing the physical properties and the biological response of calcium phosphates specifically. As schematically represented in Fig. 11, grains can be distinguished on bioceramic surfaces due to a distinct crystalline structure on the grain surface and the amorphous structure at the grain boundary [145–147].

**Figure 12.**
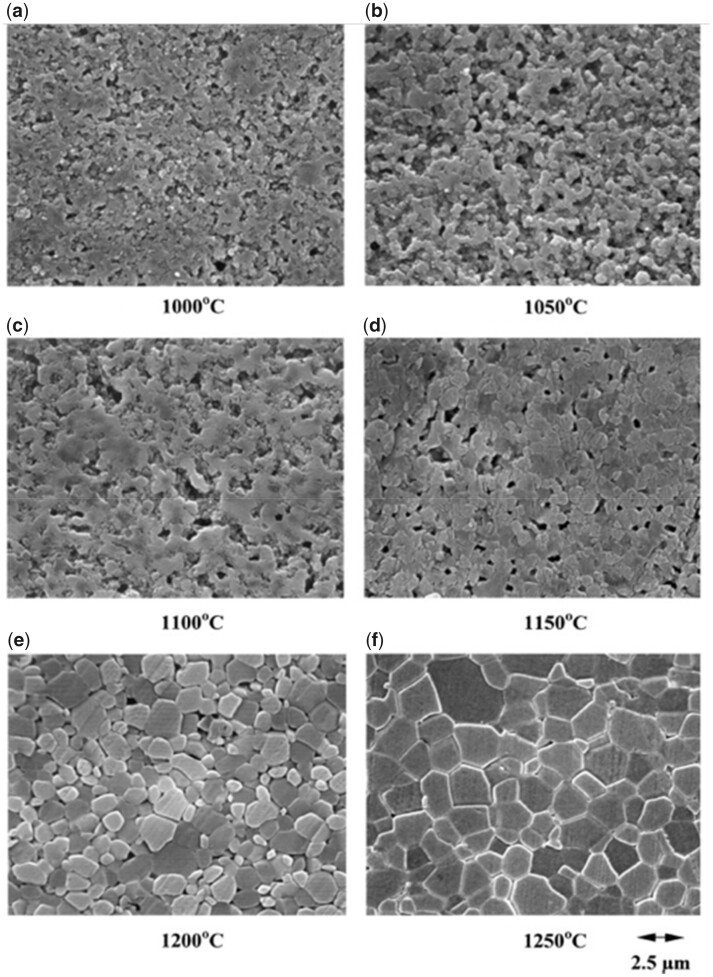
SEM micrographs of polished and etched HA surfaces sintered at various temperatures showing increasing grain size with increasing sintering temperature. Obtained from Ref. [149]. Copyright ©2000 Elsevier Science Ltd and Techna S.r.l.

One of the main factors influencing the grain size of the sintered compact is sintering temperature [148–150]. Figure 12 shows scanning electron microscopy (SEM) images of HA surfaces produced using different sintering temperatures. As seen from these images, the grain size increases with increasing sintering temperature [149].

**Figure 13.**
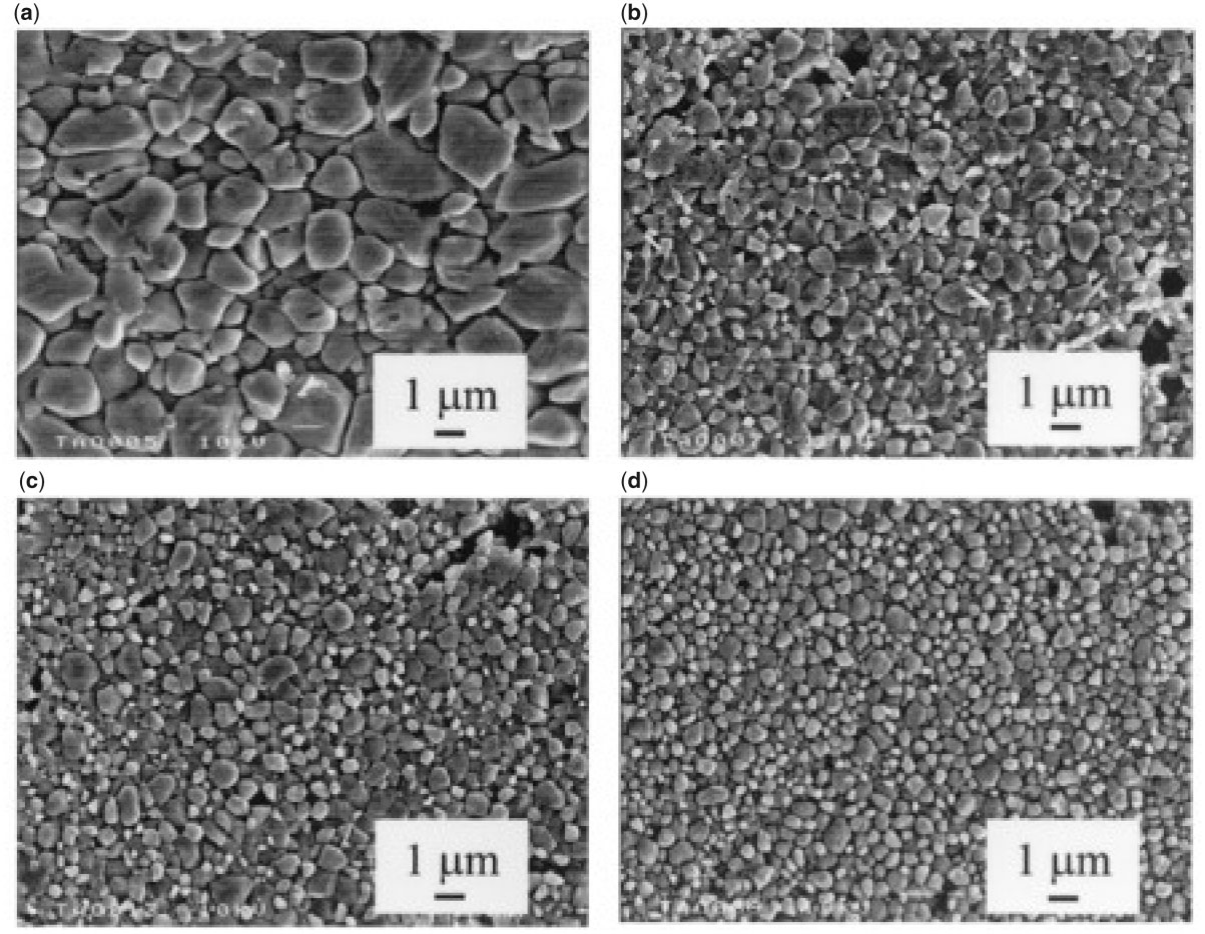
SEM images showing the effect of silicon substitution on the grain size by comparing (**a**) HA, (**b**) HA-0.4Si, (**c**) HA-0.8Si and (**d**) HA1.6Si surfaces sintered at 1200°C. Silicon-substituted HA exhibits smaller grain sizes than HA, with the smallest grain size obtained for samples with the highest silicon content of 1.6 wt%. Obtained from Ref. [143]. Copyright ©2002, the American Ceramic Society.

Changes in the chemical composition of calcium phosphate powders may also have a significant effect on grain growth. Silicon-substituted HA, for example, exhibits a significantly smaller grain size compared with stoichiometric HA. Figure 13 shows the difference in grain size between phase-pure HA and Si-HA surfaces sintered at 1200°C, with the latter group exhibiting smaller grains. The smallest average grain size was obtained for samples with the highest silicon content of 1.6 wt% [143]. According to Gibson *et al.* [143], it appears that silicon, to some extent, directly inhibits the crystal growth as evidenced by the higher activation energy of grain growth observed for these materials without showing differences in sintered density compared with phase-pure HA when a certain sintering temperature was reached. Additionally, silicon substitution was found to increase the onset temperature for sintering, which may simply mean that, at the assessed sintering temperature, the grain growth in these materials was at different stages [143].

Smaller grains increase the number of sub-grain boundaries and triple junctions which has been found to increase the solubility of the material at the nanoscale, affecting both cell behaviour and protein attachment [32, 151]. This process has been found to initiate at defects and/or grain boundaries and is suggested to be increased at incoherent boundaries without lattice continuity [32, 151]. Several studies have shown increased dissolution from Si-HA as opposed to phase-pure HA and this has been attributed, amongst other things, to the increased number of triple junctions, grain boundaries and defects on Si-HA due to the smaller grain sizes [151, 152]. The grain structure also influences solubility, with phase-pure HA exhibiting dissolution at voids on the grain surface instead of the grain boundaries as was observed for Si-HA [151, 152]. Smaller grains have also been related to the increased mechanical performance of the substrate, with Si-HA showing improved Vickers hardness, as compared with phase-pure HA [143].

#### Composition

Collagen is known for its complex supramolecular structure and is a key factor for maintaining the structural and biological balance and integrity within connective tissue. These excellent and unique properties have led to the development of advanced biomaterials using collagen type I and II to mimic the tissue at both biological and structural levels resulting in good integrity and reduced rejection of biomaterials once implanted [44].

The collagen superfamily, labelled with Roman numbers, consists of at least 29 genetically distinct variations of collagen, all differing in their chemical structure and molecular organization [44]. Collagen molecules self-associate to form higher-order structures such as fibrils and networks. Fibrillar collagen is of great interest because of its innate biomechanical stability [153–155]. Common types of collagen from this subfamily are type I, the most widespread collagen constituting the main structural protein for skin, bone and tendon, type II, with a very distinct tissue distribution found exclusively in cartilage, and type III, found in elastic tissues such as embryonic skin and lungs [155].

**Figure 14.**
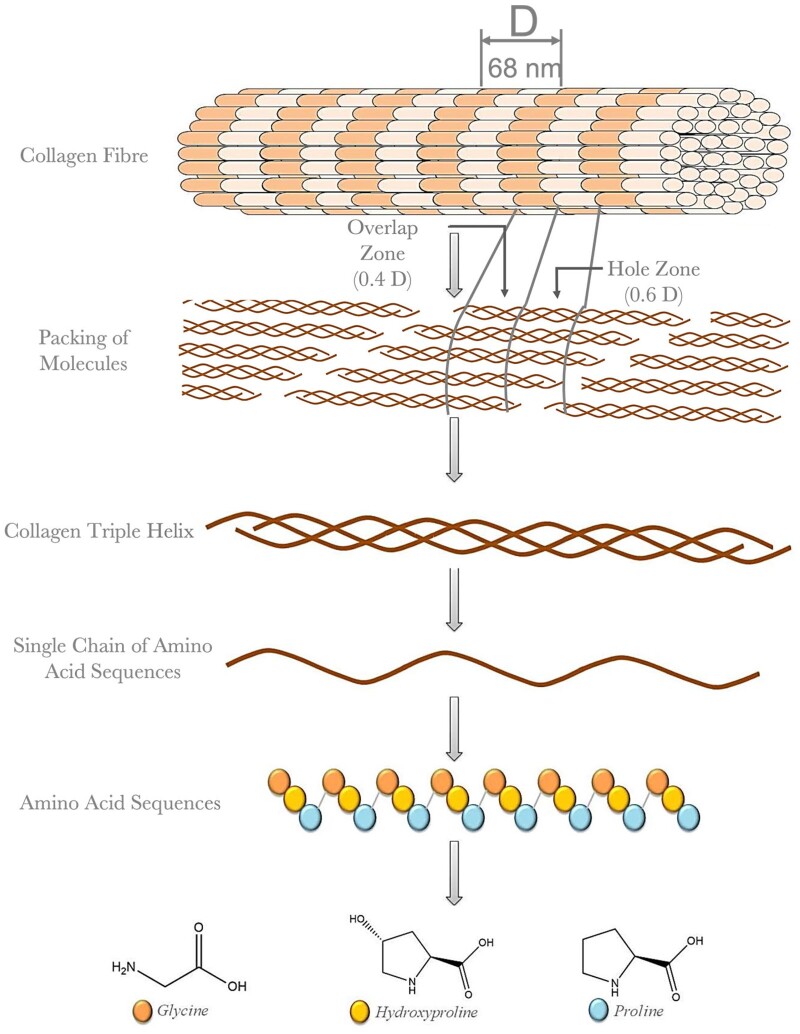
Hierarchical structure of fibrillar collagen. Collagen fibrils exhibiting low packing density hole zones and high packing density overlap zones, resulting from the characteristic quarter-staggered arrangement of single collagen molecules known as tropocollagen. Tropocollagen consists of a right-handed triple helix of three parallel left-handed polyproline II-type chains of equal length containing the repeating amino acid sequence [*Gly*−*X*−*Y*]_*n*_, with the positions X and Y being predominantly occupied by proline and hydroxyproline. Adapted and reproduced from Ref. [163].

Collagen encapsulates various glycoproteins consisting of three defining and distinct features. First, an elegant structural motif consisting of three parallel left-handed polyproline II-type chains of equal length forming a righthanded triple helix connected through hydrogen bonding, as shown in Fig. 14, illustrating collagen type I, specifically [156, 157]. While collagen type II and III are homo-trimeric, having three identical polypeptide chains, collagen type I consists of two α1 chains and one α2 chain, also known as hetero-trimeric [155]. The second characteristic of a collagen molecule, also known as tropocollagen, is the repeating amino acid sequence [*Gly*−*X*−*Y*]_*n*_ (‘Gly’ referring to the amino acid glycine), followed by the third characteristic feature X and Y, being mostly occupied by proline and hydroxyproline, respectively [156]. These features combined contribute to the structural integrity of collagen and are of key importance for extracellular matrix organization and function through exposure of cell surface receptor-recognition motifs [158].

The rigid rod-shaped molecule tropocollagen is staggered side-by-side through an entropy-driven process and separated from one another by about one-quarter of their length, with the length of each molecule being unequal to any multiple of the separation distance. As a result, a characteristic structure is formed, called quarter-staggered arrangement, consisting of a low packing density hole zone and a high packing density overlap region, first deduced by Hodge and Petruska in 1963 [159, 160]. The resulting microfibrils subsequently assemble into higher order arrangements such as fibrils, fibres and fascicles, as seen in Fig. 14 [161, 162].

Critical stabilization of collagen occurs through cross-linking either within or between microfibrils. The process of cross-linking occurs as a result of post-translational modifications such as hydroxylation of proline or lysine (Lys) residues, glycosylation of specific hydroxylysine (Hyl) residues or oxidative deamination of the e-amino groups of peptidyl Lys/Hyl [164]. While cross-linking is crucial to establish favourable mechanical properties, excessive cross-linking will lead to brittle collagen fibrils, which is a common phenomenon of ageing [156]. Besides providing structural stability, collagen is of crucial importance as a bioactive surface through the exposure of specific cell adhesion sequences. Common sequences include RGD (Arg-Gly-Asp), GLOGEN (Gly-Leu-Hyp-Gly-Glu-Asn) and GxOGER (Gly-x-Hyp-Gly-Glu-Arg), with each exhibiting a specific affinity profile for different cell surface receptors known as integrins [165]. Integrins, consisting of two non-covalently associated transmembrane subunits α and β, regulate diverse physiological processes such as cell adhesion, cell differentiation, cell migration and wound healing [165, 166]. To date, 24 different integrins have been identified. Major collagen-binding integrins are α1β1, α2β1, α10β1 and α11β1 with fibrillar collagen being more preferentially associated with the α2β1 and α11β1 integrins [167]. Collagen-binding integrins interact with ligands through the I-domain located at the α subunit. The I-domain contains a metal-ion dependent adhesion site (MIDAS), requiring a divalent cation such as Mg^2+^ to coordinate collagen integrin-binding [165, 167].

Cross-linking of collagen scaffolds using EDC/NHS also changes the chemical properties of collagen which can strongly influence the cell response. During the reaction of EDC with collagen, some of the free primary amine groups of lysine and carboxylic groups of glutamate or aspartate residues are used. These side chains, as part of the cell adhesion sequence GxOGER, are inherently involved in cell binding via β1-containing integrins. Therefore, cross-linking has a crucial impact on the cell attachment to collagen scaffolds by reducing the availability of the cell recognition motifs [133, 168, 169]. The cross-linking percentage should be closely considered to achieve the optimal trade-off between scaffold stiffness and cell response [133, 169].

In a study, Bax *et al.* [170] investigated the integrin-based binding of various cell types on collagen films and scaffolds exhibiting different EDC cross-linking degrees. While all cell types were sensitive towards EDC treatment of collagen substrates, as shown by the reduction of native-like integrin attachment with increasing EDC concentration, especially over 30%, their specific sensitivity to various cross-linking degrees, manifested by cell adhesion and cell spreading, varied between the different cell types, which can be attributed to cell type-specific collagen-binding integrins exhibiting differences in affinity to collagen ligands [170]. As different cell types ligate with collagen through different binding integrins, distinctively affecting the sensitivity to EDC cross-linking, the optimal cross-linking percentage of collagen scaffolds cannot be generalized demanding cell type-specific analysis and optimization of crosslinked scaffolds.

While a rise in cross-linking percentage has been found to reduce integrin-mediated cell binding, it has also been observed to increase the non-specific binding to collagen scaffolds [170]. As carbodiimide cross-linking not only affects the chemistry but also the stiffness and surface roughness of collagen scaffolds to which the adhesion receptors and cytoskeleton of cells are known to respond, the change in the mode of interaction can be a result of one or a combination of these factors [170]. Nonetheless, since an additional I-domain study, isolating the chemical interactions from the mechanotransductive pathways, showed a similar trend, it is most likely a result of the change in chemistry associated with the reduction in available cell adhesion sequences [170]. Although the exact mechanism behind the increase in non-specific binding with increasing EDC/NHS cross-linking remains unclear, this type of interaction has been observed to be associated with reduced cell spreading and proliferation, showing the importance of maintaining a certain degree of integrin-mediated cell binding, whilst improving mechanical stability of collagen scaffolds through carbodiimide cross-linking [170].

The crystalline structure of calcium phosphates comprises hydroxyl (OH^−^) and ${\text{PO}}_{4}^{3-}$ groups, which coordinate the surrounding Ca^2+^ ions [171, 174]. Phase-pure and stoichiometric HA exhibits a Ca/P ratio of 1.67 [145, 147]. Markovic *et al.* [172] carried out a chemical characterization of HA, which serves as a designated standard reference material (SRM), using coupled plasma mass spectroscopy. The authors found a Ca/P ratio of 1.664, deviating ∼2% from the stoichiometric value, and detected a few common trace elements with a mass fraction higher than 0.0005%, which are presented in Table 2, together with the number of ions per unit-cell [172].

**Table 2.** Characterization of trace constituents in HA-SRM detected using inductively coupled plasma mass spectroscopy

Trace constituent	Mass fraction (%)	Number of ions/HA-SRM unit cell
Al3^+^	0.0029	0.00110
Ba2^+^	0.0024	0.00018
B3^+^	0.0015	0.00142
Mg2^+^	0.0029	0.00122
Na^+^	0.0031	0.00138
Sr2^+^	0.0044	0.00051
Zn2^+^	0.0009	0.00014

Sum	0.0181	0.00595

Si	0.0150	0.00546

Adapted from Ref. [172].

**Figure 15.**
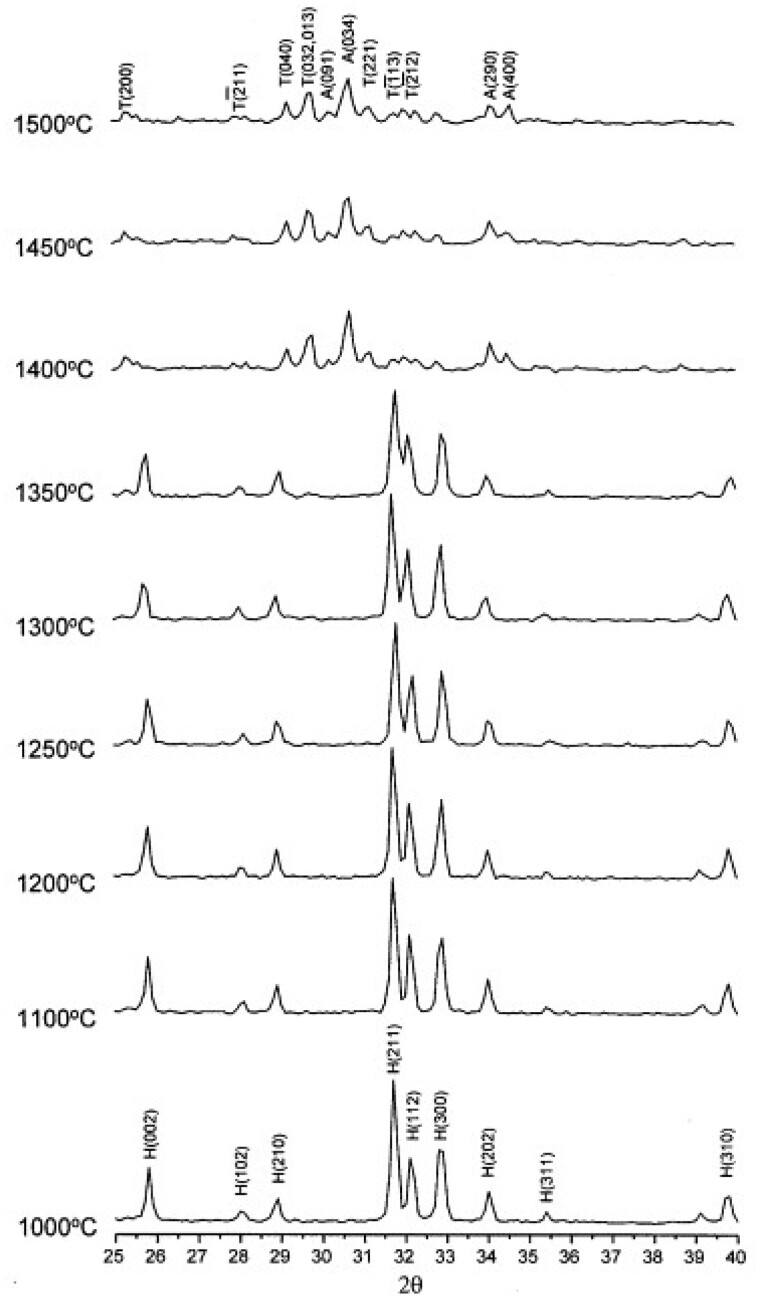
XRD patterns of hydroxyapatite powders heat treated at different temperatures. Decomposition into secondary phases was visible at a temperature above 1350°C. Obtained from Ref. [173]. Copyright ©1999, Elsevier Science B.V. (H: HAP, T: TTCP, A: αTCP).

Figure 15 shows an example of X-ray diffraction (XRD) patterns of HA heat treated at different temperatures, with all peaks identified in the samples heat treated up to a temperature of as high as 1350°C being in agreement with the HA reference pattern [173]. At temperatures beyond 1350°C, decomposition into TTCP and αTCP was identified as evidenced by the deviating XRD patterns [173]. The onset temperature for decomposition of HA powders often varies amongst different studies as it strongly depends on many factors, such as powder source, powder production method and sintering protocol, but, to date, most researchers report phase purity up to a sintering temperature of ∼1200°C [173–175]. While XRD is a powerful tool to examine the decomposition of HA into secondary phases, the transformation of HA into oxyhydroxyapatite (OHA) is difficult to detect due to overlapping peaks between the two compounds [173]. Formation of OHA results from dehydration upon sintering, which will eventually cause thermal decomposition of HA and its formation is, therefore, often monitored using Fourier Transform Infrared Spectroscopy.

#### Surface potential

Chemical compositions can strongly affect the electrical properties of a biomaterial. Being a protein, collagen consists of various ionisable residues, such as amino and carboxy groups, resulting in local differences in charge across the structure. Depending on the conformation of the collagen, these residues may be exposed or hidden and the final surface potential, therefore, depends on the number ratio of exposed amine and carboxy groups on the surface. A study found a ζ-potential of about −3 mV at pH 7 and an isoelectric point at pH 5.5 [176]. Collagen is also known to be piezoelectric, meaning that it can generate electric signals in response to mechanical stress. A study carried out using a Kelvin–Probe Force Microscope found that fibrils become more positive when strain is applied up to 10% [177]. After further stretching of the fibril up to 17%, the fibril becomes more negative again, which the authors contributed to the rearrangement of collagen in response to strain [177]. These minor changes may significantly influence how cells respond to the scaffold and should be taken into careful consideration for tissue engineering.

**Figure 16.**
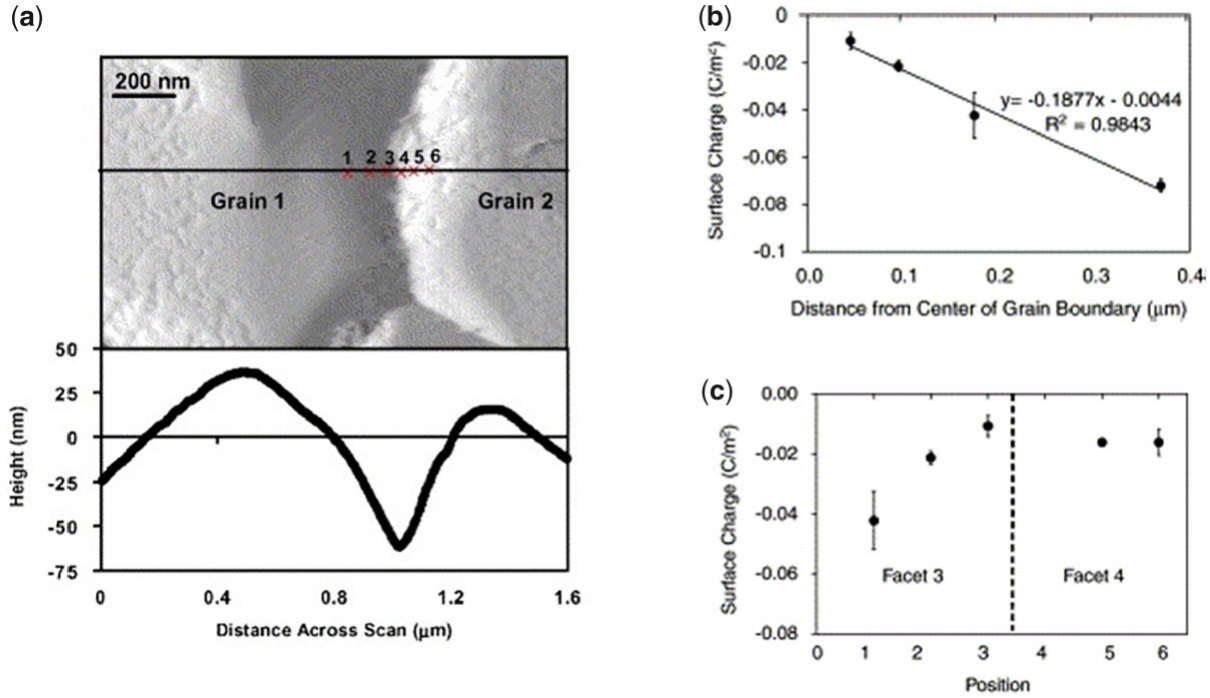
Surface charge and roughness across HA surfaces. (**a**) Contact mode atomic force microscopy deflection image using a COO^−^ terminated probe tip in fluid. The plot below shows the height profile along the solid black line in the image. (**b**) Approximation of surface charge per unit area from HRFS data fitted at each specific probe position presented in (**a**). (**c**) Linear regression of Poisson–Boltzmann fitted HRFS data showing surface charge with increasing distance from the Centre of the grain boundary. Adapted and reproduced from Ref. [178]. Copyright ©2004, Elsevier Ltd.

Vandiver *et al.* used High-Resolution Force Spectroscopy (HRFS) to measure the surface potential across HA surfaces [181]. The authors found an average surface charge of about −0.02 C/m^2^, corresponding to a negative surface potential of approximately −64 mV, which varies on a nanoscale across the surface, approaching zero towards grain boundaries (Fig. 16) [178]. This specific trend might be the result of the amorphous regions found at the grain boundary disrupting the specific ion distribution found in the crystalline regions, which exhibit an excess of ${\text{PO}}_{4}^{3-}$ ions located at the surface [178].

**Figure 17.**
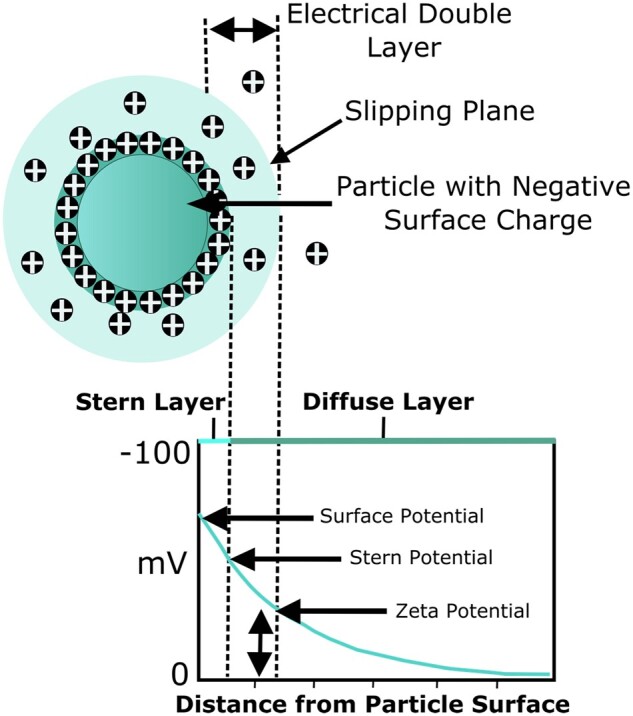
ζ-Potential of a particle with a negative surface charge adapted and reproduced from Ref. [182].

Typically, the ζ-potential method is used to measure the overall surface potential, which measures a significantly lower potential for HA surfaces, being about −35 mV as opposed to −64 mV as measured using HRFS [147, 178]. However, the ζ-potential has already been observed to be ∼30–50% of the potential measured using HRFS on zirconia surfaces which is consistent with the results obtained by Vandiver *et al.* for HA [178]. HRFS data is expected to represent the actual surface potential more closely since, as seen in Fig. 17, the ζ-potential is measured further away from the surface at the slipping plane of the electrical double layer [179]. The counter ions found in this layer and the immobilized liquid layer, known as the stern layer, cause a significant drop in potential, which results in an underestimation of the actual surface potential. HRFS data were expected to approximate the potential found at the Stern surface, which more closely resembles the electrostatic interaction sensed by a cell or protein approaching a biomaterial surface [178]. However, since the surface charge measured through HRFS is based on adhesion forces obtained for a specific interaction area, surface roughness can interfere. This effect may have accounted for the measured nanoscale variations in surface potential, known as topography-correlated artefacts [178]. Although a simple mathematical model suggested that these variations were likely to be due to electrostatic forces, direct measurements are required to prove this [178]. A follow-up study carried out on Si-HA found that, apart from inducing a more negative surface charge, silicon substitution also increases the nanoscale electrostatic, van der Waals and adhesive interactions [180]. The increase in adhesive forces was attributed to the increased polarizability of phosphate groups in Si-HA due to the reduced symmetry of silicon compared with phosphate in general [180]. Silicon substitution is also known to decrease the surface charge by replacing ${\text{PO}}_{4}^{3-}$ ions with ${\text{SiO}}_{4}^{4-}$ ions. As both proteins and cells sense and respond to surface charge, these changes may play a significant role in their bioactivity [180]. The ζ-potential at physiological pH of HA and Si-HA measured by Botelho *et al.* were approximately −50 and −71 mV, respectively [147, 181].

#### Surface wettability

Surface wettability is a measurement of SE that is dictated by the composition of the material. SE can be divided into the polar and dispersive components of the SE, with the former describing the hydrogen bonding, dipole–dipole, dipole-induced dipole and other site-specific interactions and the latter representing van der Waals and other non-site specific interactions [183]. Polar surfaces are associated with higher hydrophilicity resulting mainly from the strong contribution of hydrogen-bond accepting interactions.

In the literature, collagen is generally considered a hydrophilic protein due to the higher proportion of acidic, basic and hydroxylated amino acid residues than hydrophobic residues, with one study reporting a water contact angle of 50° for collagen extracted from rat tails [184]. However, another study on native collagen from the same source observed a contact angle of 72°, which indicates a relatively strong hydrophobicity [185]. In the same study, the contact angle was found to change with cross-linking with DHT or EDC treatment making the surface even more hydrophobic [185]. The nature of the variability in contact angle reported in literature may stem from the difference in the extraction method.

HA has been observed to be more hydrophobic than Si-HA which is believed to have resulted from the more negatively charged silicate ions substituting the less negatively charged phosphate groups, inducing a stronger interaction with the positively charged hydrogen atoms of water molecules [186]. Additionally, Vandiver *et al.* found reduced symmetry of the phosphate groups in Si-HA as the intensity ratio between the phosphate bands asymmetric stretch at 1043 cm^−1^ and symmetric stretch at 960 cm^−1^ increased from 0.440 for HA to 0.444 for SiHA which they suggest might have increased the polarizability of these surfaces as opposed to unsubstituted-HA [180]. Another surface characteristic influencing the surface wettability measured by the water contact angle is surface roughness. Generally, an average surface roughness (*R*_a_) smaller than 0.5 µm was found to have a negligible effect on the surface wetting, but, according to the Wenzel equation, increasing the *R*_a_ above this value may improve or reduce the wettability on surfaces with a contact angle larger than 90° or smaller than 90°, respectively [187]. While a strong consensus on this correlation does not exist in the literature, it is important to consider the effect of surface topography when assessing cell attachment to biomaterials [187].

### Controlling the scaffold architecture

Architectural cues such as interconnectivity, pore size and porosity of ice-templated scaffolds for tissue regeneration applications are of great importance as they have an impact on the biological response both *in vitro* and *in vivo* [188]. Obtaining scaffolds with the appropriate architecture depends strongly on the production process. Numerous scaffold production techniques have been developed over the years, with conventional methods being acid leaching, solvent casting, gas foaming, electrospinning, powder-forming processes and freeze-drying. Acid leaching and solvent casting have various disadvantages as the acid/organic solvent can be harmful to the environment, they can only be applied to a limited range of materials and do not allow control of the pore structure [189]. Gas foaming can produce high porosity and is widely applicable but does not induce the desired mechanical properties, requires high temperatures and does not allow control of the micro-scale pore structure [189]. Electrospinning creates nanofibrous constructs with high porosity and controllable alignment, but uses organic solvents, which may be toxic to the environment or humans, produces small volumes of scaffolds at a low rate and is limited in its production of complex structures including homogeneous pore size distribution [190]. Lastly, freeze-drying can be used to produce highly porous scaffolds and uses water instead of an organic solvent. However, it does use acid as a solvent for mixing the polymer, so care should be taken to wash the constructs thoroughly before use. During the freeze-drying process, both ice nucleation and crystal growth affect the morphology and structure of freeze-dried scaffolds. Consequently, any intended structural changes in the scaffold would require a specific variation of parameters targeting these factors. While freezing protocol parameters, such as set freezing temperature, set cooling rate and the addition of a thermal hold are dependent only on freeze-dryer settings, other parameters such as latent heat removal and nucleation rate also depend directly on material properties such as mould design as well as suspension composition. Various cooling methodologies based on the fundamentals of ice-templating have been reported for the design of controlled scaffold architectures from cooling in a liquid bath or a freezer to quenching in liquid nitrogen and cooling using a shelf-ramping freeze-dryer [191, 192]. The latter method allows the cooling rate and set freezing temperature to be altered separately, which is beneficial as they both influence the nucleation process and crystal growth. Many investigations only consider the set freezing temperature, neglecting the associated impact of the set cooling rate. Regardless of the cooling methodology, it is not straightforward to determine the link between a specific thermal parameter and the scaffold structure, due to the complexity of ice solidification and growth. Nevertheless, throughout the years, many studies have assessed the correlation between various factors to improve control over scaffold architecture [118, 191, 193]. Although obtaining complex hierarchical structures remains challenging, this technique allows most micro-scale control over the pore structure, distribution and size compared to the other conventional techniques [190]. As this review paper will focus on the effect or architectural features of scaffolds on tissue regeneration and, in specific, the formation of blood vessels, this technique will be explained in more detail.

#### 3D ice-templated scaffolds

Ice-templating techniques, such as freeze-casting for ceramics and freeze-drying for polymers, use the principle of ice formation to form a 3D porous structure.

**Figure 18.**
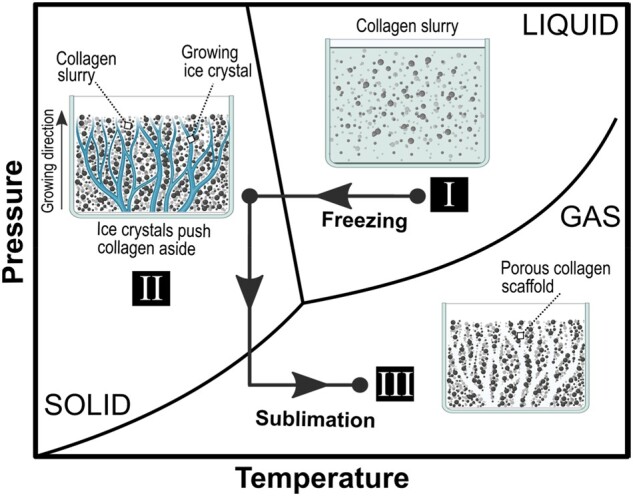
Phase diagram showing the ice solidification process and formation of a porous scaffold by means of freeze-drying. This process commences with the gradual cooling of a water-based polymer/ceramic suspension or solution below its equilibrium freezing temperature (I). the growing ice crystals exclude the polymer or ceramic dissolved as dispersed in the mould, due to the ‘self-cleaning’ nature of ice, resulting in a stable dendritic ice crystal morphology (II). the subsequent sublimation step is induced by changing the pressure and temperature of the freeze-dryer, resulting in a porous scaffold with the polymer occupying positions defined by the ice crystal boundaries (III).

The process of freeze-drying is based on the gradual cooling of a water-based polymer suspension or ceramic solution below its equilibrium freezing temperature resulting in a stable dendritic ice crystal morphology [194, 195]. Due to the ‘self-cleaning’ nature of ice, the growing ice crystal excludes the polymer or ceramic dissolved as dispersed in the mould, as shown in Fig. 18 [196]. Changing the pressure and temperature induces sublimation of the ice crystals, resulting in a porous scaffold with the polymer or ceramic occupying positions defined by the ice crystal boundaries. The final structure of freeze-dried scaffolds is, therefore, dependent on the ice crystal nucleation growth and can thus be controlled closely. Ceramic scaffolds are often subsequently sintered to improve the mechanical properties of the compact.

#### Ice solidification process

**Figure 19.**
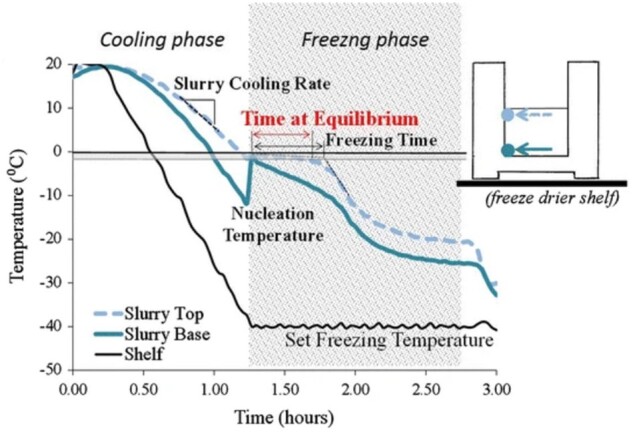
Schematic showing the temperature profile of a freezing shelf inducing characteristic temperature profiles at the top and base of a collagen slurry during freeze-drying. Obtained from Ref. [200].

In general, the process of ice solidification can be divided into two steps, nucleation and crystal growth, both having a great impact on the architecture of the resulting scaffolds. Figure 19 illustrates an example of the thermal profile of a collagen suspension in a freeze-dryer. Initially, as the temperature decreases, the nucleation of ice crystals starts, marking the onset of nuclei formation. As soon as these nuclei grow and form a stable bound, energy in the form of latent heat is released, increasing the temperature of the entire system [197]. This phenomenon, termed primary nucleation, occurs at the so-called nucleation temperature, which, for water, varies between 0 and −40°C mainly due to the stochastic nature of the nucleation process, while the equilibrium temperature remains at around 0°C [197–199]. The difference between the equilibrium and nucleation temperature is defined as the degree of supercooling [199].

The nucleation temperature is directly related to the size of the nucleus within the pure ice-water system, meaning a decrease in nucleation temperature leads to a reduction in nucleus size [201]. The rate of release of latent heat from the nuclei during primary nucleation may be faster than the heat removal rate, leading to a temperature rise, as seen in Fig. 19 [202]. From this stage, every further nucleation process and crystal growth appear at a higher temperature and is known as secondary nucleation, which is believed to be responsible for the final crystal structure [198]. After complete solidification of the suspension with all the latent heat being removed from the system, the slurry temperature decreases further [198].

Although nucleation is a stochastic process, it has been reported to play an important role in determining the final architecture of ice crystals [203]. Variations in nucleating agents, solute size and type, and the set cooling rate have been shown to affect the nucleation temperature and, thus, the final structure of the produced solid [204–207].

##### Set cooling rate

The cooling rate influences the structure of ice-templated scaffolds by influencing the start of the nucleation process [191]. Yuan *et al.* [191] observed a shift from isotropic to anisotropic morphology of alginate and cellulose-based scaffolds when altering the freezing protocol from ramping to −80°C at a rate of 0.83°C/min to quenching. A similar phenomenon was observed by Searles *et al.* [207], where increasing the cooling rate above a particular value resulted in a shift from freezing by global supercooling to directional solidification, which led to the hypothesis of the existence of a critical cooling rate. A recent study by Wang *et al.* [208] on alginate scaffolds showed that an increase in cooling rate from 0.64 to 0.68°C/min resulted in an increase in the degree of supercooling and a decrease in pore size. This article interpreted this phenomenon as being related to the existence of a critical nucleation radius below which ice crystals are unstable and, as a result, melt, in a process known as Ostwald ripening. This critical radius decreases with increasing supercooling allowing smaller nuclei to survive resulting in more and smaller pores within the final scaffold structure [208]. Therefore, it can be theorized that a set cooling rate indirectly affects the pore size by altering the degree of supercooling. A similar trend was observed for HA scaffolds freeze-cast at a temperature of −100°C, where an increase in cooling rate from <1°C/min to >5°C/min resulted in finer pores [209]. Nevertheless, O’Brien *et al.* [210] found that a quenching technique with a measured average cooling rate of as high as 4.1°C/min increased the mean pore size and worsened the overall pore homogeneity, as the very fast cooling induces a large temperature gradient across the suspension, which was virtually absent when using set cooling rates.

##### Set freezing temperature

While an isolated influence of the set cooling rate on the scaffold structure should be treated with caution, a direct and independent impact of the set freezing temperature has been demonstrated. In a study, a decrease in set freezing temperature from −10 to −40°C in a collagen-glycosaminoglycan suspension with a constant cooling rate of 1.4°C/min was found to decrease the pore size from 150 to 95 µm [118]. Moreover, Haugh *et al.* [211] decreased the freezing temperature of a collagen-glycosaminoglycan suspension from −10 to −70°C and revealed that a decrease in the set freezing temperature of the slurry below −50°C does not result in a further significant decrease in pore size below 90 µm. The author justified this trend by stating that below the critical temperature of −42°C, also called glass transition temperature, the viscosity of the frozen slurry increases, reducing water diffusion to ice crystals and, thus, any further temperature drop does not affect the pore size [211]. Overall, smaller pore sizes occur with a larger supercooling as the nucleation rate increases while the crystal growth rate decreases.

##### Thermal hold

Another significant parameter for altering the final structure is the addition of a thermal hold to the freezing cycle. Adding an annealing time influences not only the pore size but also the overall homogeneity of the pore structure [212]. Haugh *et al.* investigated the annealing effect on 0.5 wt% collagen scaffolds and demonstrated that an initial increase in the thermal hold at −10°C from 0.25 to 6 h decreased the pore size. A further increase in the thermal hold subsequently increased the pore size with a peak reached at 18 h, resulting in a 40% enlargement as compared with 0.25 h. No significant variation in pore size could be observed beyond this point [211]. The authors hypothesized that this trend is due to the previously mentioned Ostwald ripening. It is believed that the diameter of small ice crystals below the critical size will decrease over time as a result of the melting process, causing the initial decrease in pore size. After elimination of the small crystals, the remaining ice crystals will grow bigger owing to a reduced viscosity resulting in increased heat diffusion, which occurs as a consequence of the added annealing step [211].

Extending the freezing cycle has also been shown to affect the structure of the produced scaffolds. A study, performed with a 1 wt% chitosan slurry, showed that after a 16-h freezing time at −20°C the isotropic structure of the scaffolds changed from initially equiaxed and interconnected pores (at 1, 4, 6 and 8 h) to more aligned and highly disconnected pores (at 16 h) [213].

##### Mould design

The mould material, shape and form can also influence the properties of the produced scaffolds, including anisotropy and homogeneity of pores.

Mould type provides a possibility to alter the global or directional ice crystallization as it determines the crystal growth direction and imposes a temperature gradient by altering the temperature transfer [214]. For the production of aligned structures, it has been shown that as the nucleation process starts, the temperature of a portion of the suspension has to be above the equilibrium temperature, offering a temperature gradient [214].

Various mould materials have been investigated by Davidenko *et al.*, revealing the influence of the type of material on the scaffold structure. While the homogeneous and isotropic architecture was achieved using moulds comprised of one material exhibiting an even wall thickness throughout, anisotropic structures were generated using moulds with more than one material with- and without uneven base thickness [215]. The principle of this mechanism is attributed to the established temperature gradient within the slurry inside the heterogeneous moulds when nucleation takes place as the crystal growth is preferred along the gradient. In addition to the induced anisotropy, an increase in pore sizes was observed along the thermal gradients due to the increase in cooling temperature and decrease in cooling rate, as a result of the uneven wall thickness [215].

##### Time at equilibrium

Another parameter influencing the collagen scaffold architecture, in particular the pore size, is the so-called time at equilibrium introduced by Pawelec *et al.* [193]. This thermal parameter describes the time a slurry spends at the equilibrium freezing temperature where most crystal growth occurs [193]. Extending the time spent at equilibrium from ∼1500–4700 s at the top region of the scaffold for a 1 wt% collagen suspension, by increasing the freezing temperature from −40 to −20°C, has been shown to raise the pore size from 142 to ∼170 µm [193].

##### Suspension composition

Variables within the polymer or ceramic slurry can have an impact on the structure of the produced scaffolds, such as solute concentration and additional macromolecular components in the prepared suspension. Choosing various types of additives and using different concentrations lead to structural changes in the ice-templated scaffolds. The precise effect of these variables is, however, not always consistent [216].

While the addition of a solute into the suspension reduces the equilibrium freezing temperature, it also directly imposes faster and more heterogeneous ice nucleation, which together greatly lowers the degree of supercooling and subsequently affects the pore size. Therefore, changing the composition affects the structure simultaneously on various levels making it difficult to control specific parameters [192, 197]. Solutes, such as salts and acids are often used for scaffolds produced for tissue regeneration to adjust the pH of the suspension. A study found that increasing the acetic acid concentration in collagen suspensions from 1.5 to 3.8 wt% resulted in larger pore sizes [195]. Adding salt to a silk fibroin solution, results in change in the pore interconnectivity as well as the mechanical and swelling properties of the produced scaffolds [217].

Increasing collagen concentration within a suspension may be expected to increase the possible sites for ice crystal formation leading to a higher freezing temperature and a reduction of supercooling, and thus larger pore sizes [162]. Previously published data showed that increasing the collagen weight per cent in a suspension from 0.25 to 1 wt% leads to an increase in scaffold pore size [162]. The small pore size of 0.25 wt% scaffolds was attributed to the weak mechanical properties, resulting in collapsing of the scaffold [162]. Conversely, Madaghiele *et al.* [218] reported that increasing the collagen content from 0.5 to 2 wt% generated smaller pore sizes owing to the reduced diffusion rate of water molecules as a result of increased viscosity. It could, however, be possible that a critical concentration exists, depending on various factors, below which the effect of collapsing of the scaffold due to weak mechanical properties on pore size counteracts the impact of the reduced viscosity, inducing a smaller pore size. Nevertheless, another study contradicting both findings reported that no significant change in pore size could be observed as collagen concentration increased from 1 to 2 wt% [219].

In the case of ceramics, additives such as gelatin, polyethylene glycol, glycerol and polyvinyl alcohol are added to the colloidal solution to serve as a binder, which aids the formation of a homogeneous slurry [220]. As for the case of polymers, such additives can affect the pore size and morphology of the scaffold. Additionally, a foaming agent can be added which produces gas bubbles inside the solvent by rotating the suspension or blowing an inert gas. Depending on the liquid-gas interface, modifiers can then be used to stabilize the foam, which, in turn, determines the pore size and its distribution as well as the morphology of the final construct [220]. As ceramics are thermally stable, it is also possible to substitute water for a liquid with a higher melting temperature, such as camphene and tert-butyl alcohol, circumventing the need for very low temperatures and pressure. In the case of tert-butyl alcohol, the pores will be unidirectional hexagonal due to the characteristic morphology of the ice crystals after freezing being dendritic crystal straight prisms. Using these solvents will also allow for the application of very diluted ceramic solutions which aids the process of obtaining highly porous scaffolds with high interconnectivity [220].

## Biological response to biomaterials

Both physical and electrochemical scaffold properties greatly influence how biological systems respond to the biomaterial. Over the years, many studies have improved our understanding of these processes, but much remains unknown. In particular, contradicting findings have made it complicated to obtain a generalized understanding of the mechanisms and have shown the complexity of these events. It is, therefore, important to critically assess papers, as minor differences in the experimental protocol may account for major differences in outcome.

### Events at the biomaterial interface upon implantation

**Figure 20.**
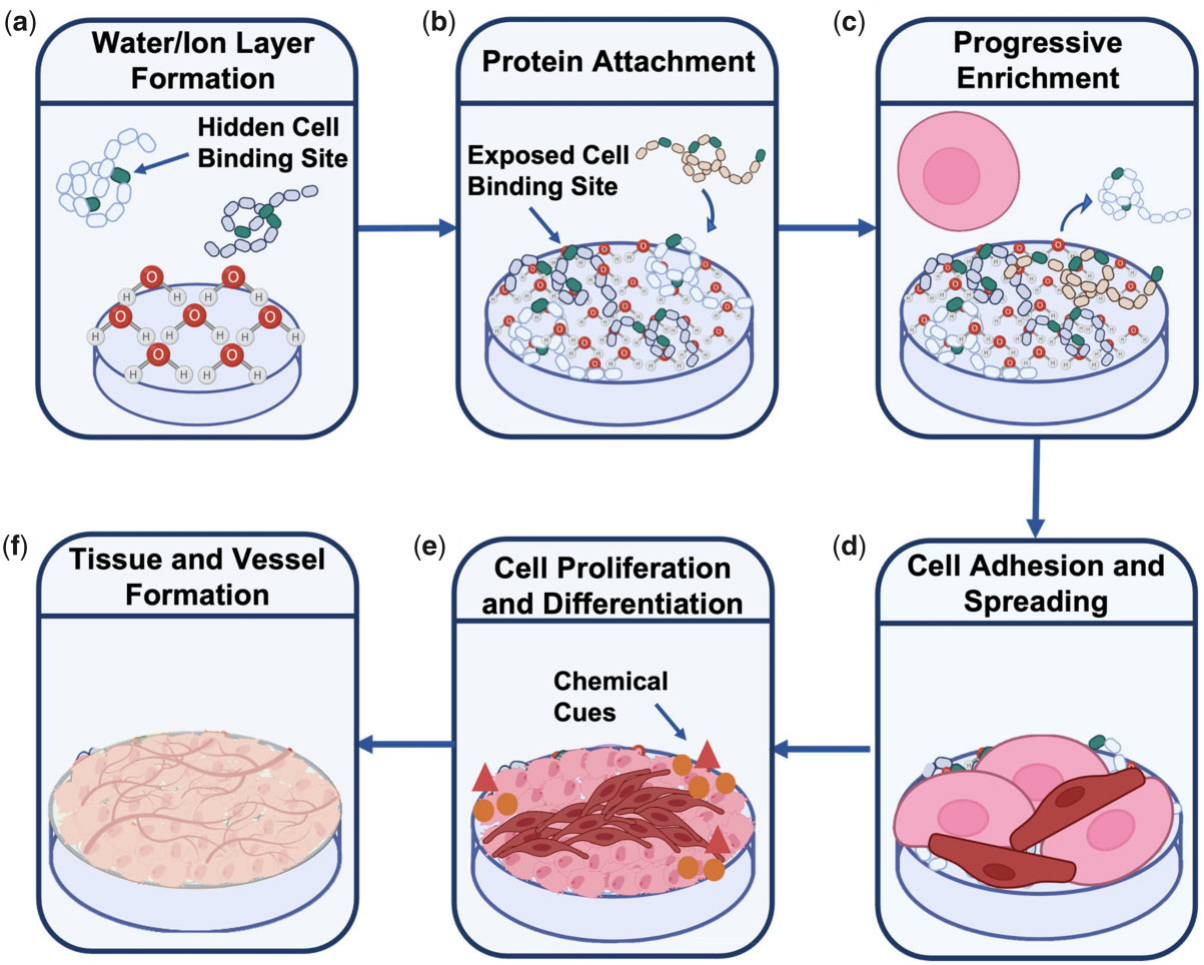
Schematic of events occurring at the biomaterial interface upon implantation. Firstly, water molecules and specific ions attach to the surface (**a**) followed by protein attachment (**b**) and progressive enrichment (**c**). The final nature of the protein layer is what is essentially sensed by the cells that subsequently reach the surface (**d**). After appropriate cell attachment, cells begin to proliferate and differentiate and will eventually release various chemical cues such as VEGF or HIF (**e**), directing cells towards tissue and vessel formation (**f**). The overall evolution of the tissue formation strongly depends on the characteristics of the attached hydration and protein layer.

Upon implantation of a biomaterial, water molecules and specific ions are attracted to the surface, creating a hydration layer (Fig. 20a). Rapidly after this stage, proteins found in the blood and ECM attach to the surface, providing essential cell-binding sites and extracellular instructions (Fig. 20b). After this stage, competitive protein exchange may occur, depending on the biochemical and electrical affinity of a protein to the surface and the abundance of a protein, known as the ‘Vroman effect’ or progressive enrichment (Fig. 20c) [221]. Adsorbed proteins are generally replaced by larger and conformationally flexible proteins enabling stronger attachment, but the precise mechanism is still unknown [221]. The final protein composition, the amount and conformation are directly influenced by the composition of the hydration layer and the biomaterial, as a result of the van der Waals and electrostatic forces, hydrogen bonding and hydrophobic interactions involved in protein adsorption [116]. Protein attachment consequently influences the final cell attachment by exposing (or hiding) specific cell-binding sites [222]. The overall process occurs within milliseconds after contact with biological fluids, which is why cells will generally only sense the biomaterial indirectly (Fig. 20d). At the final stage, cells will begin to proliferate, differentiate and drive tissue and vessel formation depending on the nature of the cell attachment and the angiogenesis-related cues given by the cells, such as VEGF and hypoxia-inducible factor (HIF) 1α (Fig. 20e and f) [222]. These chemical cues can direct ECs migration, proliferation and ultimately formation of tubular structures.

**Figure 21.**
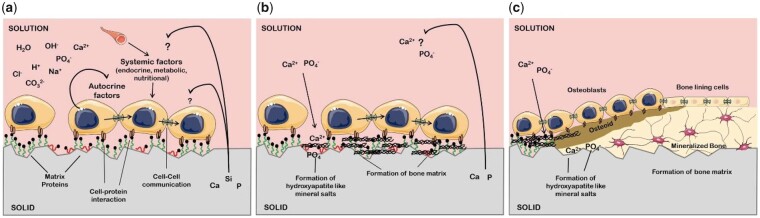
Schematic representation of cell–material interactions mediated through protein attachment (**a**) and bone formation resulting from dissolution of ions from the biomaterials and precipitation reactions onto the surface creating hydroxyapatite-like mineral salts (**b**), which will eventually promote formation of a new bone matrix (**c**). Adapted from Ref. [31]. Copyright ©2011, Z. Mladenovic.

Of all the various proteins found in the ECM, collagen-I, laminin, fibronectin (FN) and vitronectin (VN) have been found to be most involved in OB adhesion and blood vessel formation [223–225]. These proteins contain integrin-binding domains such as arginine–glycine–aspartate (RGD), which has been found to be beneficial for the attachment of cells in general and, in particular, of human MSCs that can differentiate into OBs [224]. When cells approach the surface, they initially interact with the biomaterial surface through short-range physicochemical interactions. Shortly after, integrins located on the membrane recognize the binding sites presented by adsorbed proteins. They cluster together, triggering cytoskeleton reorganization, resulting in stabilization of stress fibres and focal adhesions crucial for healthy cellular functioning, as schematically represented in Fig. 21a [31, 7^226^]. The exact mechanism by which these proteins direct ECs to form vessels is still unclear, but various theories have been proposed based on several *in vivo* observations. Knock-out of collagen-I in mice, for example, was found to result in unstable vessels [225, 227]. While vessel membranes contain collagen-IV and laminin but no collagen-I, it is proposed that exposure of ECs to collagen-I is an indication of degradation of the basement membrane of blood vessels, which may activate these cells to form new blood vessels [225, 227]. Furthermore, laminin has been found to promote migration of ECs and a lack of FN was found to induce primitive blood vessels, which indicates its role in angiogenesis [228, 229]. FN, specifically, has been observed to exhibit two domains that regulate the activity of VEGF, which is believed to play a key factor in its ability to modulate vessel formation [230].

As the incubation time increases, the biomaterial may begin to degrade, resulting in either small (poly-)peptides consisting of [Gly–X–Y]_*n*_ for collagen or ion dissolution for HA [231]. Ion dissolution of HA involves the release of Ca^2+^ and ${\text{PO}}_{4}^{3-}$ ions into the ECM, resulting in the formation of a calcium-phosphate layer (Ca-P layer) onto the biomaterial through precipitation reactions which aids the mineralization process necessary for bone healing (Fig. 21b and c) [31, 152]. Dissolution is often suggested to be one of the mechanisms underlying the bioactivity of HA, as the formed carbonate-containing apatite layer onto biomaterials makes an ideal surface for cells to adhere, grow and form new bone, enabling successful osseointegration *in vivo* [152]. Both collagen and HA degrade into physiologically tolerable compounds and are, therefore, biocompatible.

### Effect of surface properties on bone tissue regeneration and angiogenesis

Studying the protein/cell attachment and vessel formation and understanding how these can be modulated to suit the requirements for healthy bone tissue regeneration is key for improving tissue engineering outcomes. These biological processes are, however, complex and depend on numerous parameters, including properties of the protein such as size, charge, structure, stability and unfolding rate as well as physical and chemical properties of the biomaterial, such as surface wettability, geometry, surface roughness, grain size and boundaries and surface charge [232]. The biological response to a scaffold is dictated by the surface properties, due to the intricate interaction between a biological system and the surface of a material. As a biological system interacts at a nano-, micro- and macroscale, characteristics at each level are important to consider when assessing the bioactivity of a biomaterial. Various features are strongly intertwined, making fundamental research on the biological response to scaffolds complicated with often contradicting trends reported in the literature. Nonetheless, some general trends can be extracted, and comprehensive discussions on the observed correlations provide a strong fundament for continued research in this area.

#### Mechanical properties

Stiffness of a biomaterial is not only crucial for the stability of an implant, it is also known to direct cell morphology, proliferation, migration and differentiation. Generally, obtaining the optimal scaffold stiffness is not as straightforward as simply mimicking the stiffness of the target tissue. Collagen scaffolds with stiffness as low as 1.25 kPa, much lower than the natural stiffness of bone (≈1 GPa), have been found to induce appropriate OB proliferation, while even more compliant substrates with stiffness of ∼0.45 kPa were observed to enhance the maturity of OBs to a mineralising phenotype [233]. Olivares-Navarrete *et al.* [234] also recognized that for appropriate OB attachment ‘the harder, the better’ cannot simply be assumed, reporting significantly impaired hOB behaviour on substrates with a Young’s modulus closer to that of the cortical bone. A possible explanation for these observations might be the naturally dynamic ECM stiffness for OBs during bone formation, starting with softer, pre-mineralized collagenous bone (30–100 kPa), followed by ECM stiffening over time as a result of OB matrix secretion and mineralization [235, 236]. Stiffening of collagen-GAG scaffolds has also been reported by Keogh *et al.* [233] over a 6-week culturing period which they ascribed to a combination of cell-mediated contraction and ECM deposition and mineralization. To date, no consensus exists about the optimal stiffness of scaffolds for OBs and as cell behaviour also strongly depends on substrate type, structure and various other conditions, generalization of this scaffold parameter may not be feasible [233–235]. In terms of angiogenesis, it was found that stiffer polydimethylsiloxane substrates (PDMS), moving from 6 to 135 kPa, up-regulates the production of angiogenesis-related GFs in HUVECs [237]. However, it is important to note that the ECs were co-cultured with a lung cancer cell line, so the observed process may deviate from angiogenesis in healthy tissue. Another study found enhanced anchorage and cell survival of HUVECs on stiffer PDMS substrates with a Young’s modulus of 2000 kPa compared with soft surfaces with a Young’s modulus of 50 kPa [238]. They stated that the improved anchorage through enrichment of lamellipodia and filopodia in the initial hours of culture possibly contributes to a better cell adherence and spreading which, in turn, induces enhanced cell viability on the long term [238]. An appropriate surface stiffness can provide the cells with a strong foundation to proliferate after which the cell confluency increases and signals from surrounding cells become more important that signals from the substrate [238]. Stiffer surfaces (*E* = 195 kPa as opposed to *E* = 15 kPa) were also found to more strongly stimulate the paracrine pathways of adipose-derived stem cells that promote vessel-like structure formation of ECs [239]. The authors observed a signalling cascade induced by surface stiffness that ultimately culminates in vessel-like structure formation [239]. Upon cell attachment changes in vinculin distribution were observed on the cell membranes, which is a molecule in focal adhesion plagues that establishes attachment of cells to the ECM. Subsequently, intracellular β-catenin signalling is activated which involved binding of β-catenin downstream proteins to the promoter of VEGFA, which induces angiogenesis [239].

#### Surface wettability

Surface wettability is an important parameter influencing protein and cell attachment, and therefore vessel formation, but inconsistent results regarding the effect of this property on the biological response have been reported throughout the literature. Initially, there appears to be a general understanding that the driving force for protein attachment is higher on hydrophobic surfaces as opposed to hydrophilic surfaces [240–242]. This trend can be explained through the second law of thermodynamics, describing that in a spontaneous process entropy never decreases. Upon implantation, a hydrophobic biomaterial will cause the water molecules near the surface to associate more strongly with themselves, which is an energetically unfavourable process due to the loss in entropy [240]. As a result, hydrophobic moieties of the protein will associate with the biomaterial surface forcing out water molecules from their structure, increasing the entropy to re-establish the energetically favourable conditions [240]. Interestingly, however, in some cases, papers have found enhanced protein adsorption on hydrophilic surfaces [243, 244]. These contradicting findings may indicate that other factors are, to a greater extent, responsible for a protein’s affinity to a particular surface, such as the type of protein or its structural stability or surface topography [242, 245]. For example, Webster *et al.* [243] found increased VN adsorption on more hydrophilic nanophase ceramics, but these surfaces were simultaneously associated with smaller grains. As grains enhance the surface roughness of samples, which increases the specific area for protein attachment, this surface property may have actually been the driving force for increased protein attachment [243]. Another study found that FN attachment without the competition of other proteins was higher on both hydrophilic and hydrophobic surfaces compared with surfaces with moderate hydrophilicity. However, when albumin was added to the system, FN attached better to hydrophilic surfaces. Albumin, on the other hand, was more attracted to hydrophobic surfaces, both with and without competition from FN [244].

In terms of cell attachment, a consensus exists in the literature regarding their response to surface wettability, with overall a lower cell attachment measured on hydrophobic surfaces [185, 223, 246]. Altankov *et al.* [246] believe the reduced cell attachment on hydrophobic surfaces to be a direct result of the strong interaction between the proteins and the surface, preventing reorganization of, in their case, FN into ECM-like structures, a process they suggest to be necessary for normal cell function. Other explanations could be the stronger unfolding or denaturation of proteins on hydrophobic surfaces, resulting in unfavourable positioning of the cell-binding sites, or the inability to displace proteins due to the stronger association with the surface, preventing progressive enrichment of the biomaterial surface with new proteins, known as the previously mentioned ‘Vroman effect’ [120]. In general, it is not only the amount of protein that affects the cell attachment but also the reactivity resulting from a protein’s orientation and conformation. Mitsi *et al.* [247], for example, found that the VEGF domains on FN were exposed only on hydrophilic surfaces where FN assumes an extended conformation which could promote better neovascularization. Additionally, hydrophilic rough titanium surfaces were found to enhance the production of angiogenic factors in EPCs, but the authors do believe that surface roughness played a more substantial role than wettability [248]. Nevertheless, another study carried out in co-culture with OBs found that the proliferation of HUVECs was more strongly influenced by the hydrophilicity of the surfaces rather than their roughness and found that the proliferation was enhanced on hydrophobic titanium surfaces compared with the hydrophilic counterparts [249]. These results contradicted mono-culture findings obtained by the same authors, which showed improved proliferation of HUVECs on hydrophilic smooth titanium surfaces, indicating the importance of co-culture studies which provide a closer approximation to the *in vivo* environment [249]. Overall, the exact influence of surface wettability on protein and cell attachment, and their subsequent effect on angiogenesis, remains unclear but from these papers, a general trend can be extracted stating that although hydrophobic surfaces generally provide a stronger intrinsic driving force for protein attachment, proteins may still, in some cases, possess a higher affinity for hydrophilic surfaces. Additionally, cells prefer hydrophilic surfaces, possibly due to the difference in nature of the protein attachment.

#### Geometry and surface roughness

Roughened surfaces are generally associated with increased protein adsorption and improved cell attachment because of easy entrapment of proteins in the grooves, increased specific surface area for adhesion and favourable integrin clustering, allowing mechanical anchoring of cells [251–253]. While this seems straightforward, contradicting results have been reported, which appear to demand a deeper understanding of the term surface roughness [226, 251].

**Figure 22.**
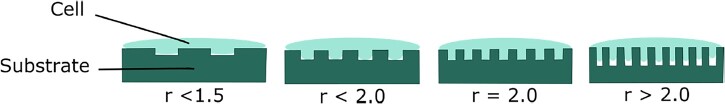
Schematic representation of cell–biomaterial contact on surfaces with different roughness ratios. Adapted and reproduced from Ref. [226]. Copyright ©2021, The Royal Society of Chemistry.

Firstly, a study revealed that it is necessary to discern between organized and disorganized substrates, as they observed that disorganized rough Ti6Al4V alloy surfaces were associated with reduced cell adhesion and proliferation compared with the smooth counterpart [253]. Secondly, the stage of cell attachment and the type of cell response measured could have a significant impact on the outcome of the analysis. A study found better proliferative capacity and alkaline phosphatase activity but lower osteocalcin production for adult jaw bone cells on smooth titanium surfaces (polished) compared with plasma-sprayed rough surfaces, at an early stage [254]. However, they do stress that, in the long term, rough surfaces performed better overall, which may, in the end, provide more favourable conditions for clinical applications [254]. Furthermore, Majhy *et al.* [226] identified a critical surface roughness ratio (*r*), a parameter indicating the ratio of actual contact area to the projected area parallel to the plane of a surface. They found that an increase in roughness ratio up to *r* = 2 increased cell growth and proliferation on polydimethylsiloxane surfaces, while higher roughness ratios induced a reduced cell response for HeLa and MDA MB 231 cells. As seen in Fig. 22, if the roughness ratio is too low, the cell membrane is unable to fully penetrate the grooves, reducing the specific surface area available for cell attachment [226]. When a particular roughness ratio is reached, the cell membrane almost completely covers the surface area in the grooves, allowing for a favourable positioning of the integrin clusters necessary for the formation of mature focal adhesions [226]. However, when a critical roughness ratio is exceeded, the limited elasticity of the cell membrane prevents it from fully penetrating the grooves, resulting in a reduced cell attachment [226].

Lastly, nano-, micro- and macro-roughness features interact with various cell components at different levels, eliciting a distinct cell response. Such features may naturally exist as a result of the grain surface/grain boundary structure, or can be induced through surface patterning. Nanoscale features are believed to more strongly influence the formation of focal adhesions, being within the range of 5–200 nm, and most closely resemble the natural tissue morphology [250]. Furthermore, as proteins are in the nanometer range, nanoscale characteristics may influence their attachment and conformation, which, in turn, affects cell attachment [243, 250]. Overall, little is known about the influence of nanoscale features on protein attachment. With different trends being reported in literature, it has been proved complicated to generalize the correlation between this surface characteristic and the biological response. On the one hand, Cai *et al.* [255] reported that on titanium films, differences in nanoscale roughness with a root mean square between 2 and 21 nm had little influence on the protein adsorption and cell growth. On the other hand, Webster *et al.* [243] concluded that various nanophase ceramics associated with a surface roughness above 17 nm induced improved protein and cell attachment compared with conventional ceramics with a roughness between 10 and 17 nm. While both papers revealed an increase in wettability with increasing surface roughness, the samples used in the former paper were considerably more hydrophobic than the samples used in the latter study, which may have contributed to the contradicting findings [243, 255]. While microscale roughness is also known to influence cell adhesion and cell morphology, macroscopic roughness generally only affects cell morphology as cells have enough space to attach between the macroscale features [250, 251]. On collagen films, microscale roughness was found to be affected by the degree of cross-linking, with this treatment significantly reducing the roughness of collagen films [256]. The authors also found a significant reduction in myoblast cell coverage of the samples and they hypothesize that this may be related to the lower surface roughness [256]. However, since cross-linking is strongly associated with a reduction in available cell binding sequences on collagen, there are multiple factors at play here. They also mention that samples of different compositions may not be directly comparable, with gelatin films, being more smooth, inducing increased cell attachment compared with collagen films [256]. Overall, most of the proposed correlations remain under debate.

Apart from the surface roughness modulating the cell adhesion, surface geometry (including shape, size and spacing between features) also has a strong influence on the morphology of the cells through contact guidance. Grooved surfaces have been shown to result in cell alignment, which can be beneficial for clinical applications as similar structures are also observed in the natural ECM of tissues such as heart myocardium [257]. A study carried out by Chesmel *et al.* [258] found that 5 µm grooved polystyrene surfaces induced cell alignment of osteoblastic cells but grooves of 0.5 µm were unable to impose this change in cell morphology. On alumina surfaces, it was observed that human MSCs also responded to narrow grooves and ridges of about 10 µm, resulting in elongated cell morphology, but it was found to be the surfaces with wider grooves of about 50 µm that induced the most suitable conditions for osseointegration as evidenced by the enhanced formation of osteoid matrix nodules [259]. Vascular smooth muscle cells and ECs are also well-known to favour aligned grooves or other aligned surface features, such as nanofibres over randomly arranged surface characteristics as they aid cell alignment and directional growth [260]. This phenomenon has been attributed in literature to reassembling of the actin cytoskeleton and inhibition of inflammation as a result of the alignment at the surface of the biomaterials [260]. HUVECs, HDMECs, human saphenous vein ECs and human aortic ECs were all found to migrate along the long axis of the anisotropic surface features and its effect was most pronounced on topographic ridges >800 nm, with the largest measured size being 4000 nm [261]. The authors also found that the rate of cell migration varied between the different groove and ridge sizes, which they believe may be an indication that topographical cues may be involved in the process of neovascularization *in vivo* [261].

**Figure 23.**
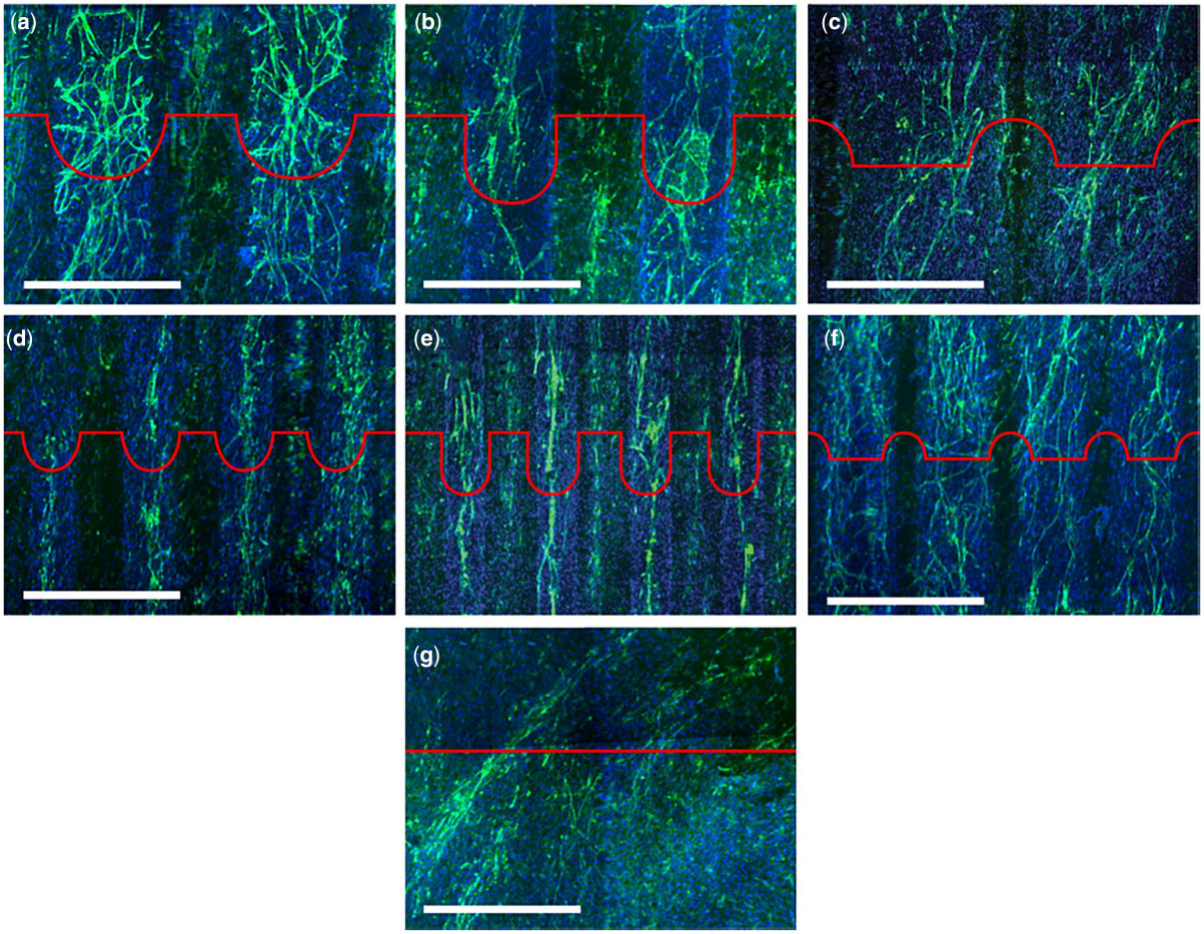
Confocal images of EC and OB co-cultures on HA surfaces showing self-assembled microcapillary-like structures on (**a**) large concavities, (**b**) large deep concavities, (**c**) large convexities, (**d**) small concavities, (**e**) small deep concavities, (**f**) small convexities and (**g**) flat surfaces as indicated by the red profiles. Obtained from Ref. [175]. Copyright ©2019, Gariboldi *et al*.

Gariboldi *et al.* [175] found that architectural features on biphasic HA/α-TCP surfaces can be used to control blood vessel formation, with small concave grooves functioning as a biomimetic cue for localization and orientation of tubular structures, as seen in Fig. 23. Vessel-like structures were observed to concentrate in concavities or between convex surface features, with the latter geometry preventing network continuity [175]. The substrate cues are suggested to mimic the Haversian canals which surround blood vessels in healthy bone and may thus induce the formation of vessel-like structures on biomaterials [175]. Furthermore, in the concavities and areas between convex features pro-angiogenic factors may build-up which directs angiogenesis towards these areas and/or the edges may simply serve as a biomechanical barrier [175].

Furthermore, Rouahi *et al.* [262] argue that increased microporosity of HA samples may also significantly contribute to change in cell morphology and improved cell adhesion due to increased protein adsorption. The authors compared the bioactivity of relatively dense and microporous HA discs and found significantly enhanced protein and initial OB cell attachment, but reduced cell proliferation on the microporous discs. They suggested that the increased surface roughness due to microporosity may have enhanced the protein attachment onto these discs, which subsequently improved the initial cell attachment. Additionally, cells appeared to have more intimate contact with the microporous discs, potentially due to the capillary forces, which may have hampered the cell proliferation at later stages [262]. While another study did find a comparable effect of microporosity of HA samples on cell proliferation, this surface characteristic was not observed to influence initial cell attachment [263]. Since similar microporosity percentages were reported by both studies, it may have been caused by the different disc production processes, with Rouahi *et al.* including a polishing step for the dense samples [262, 263]. As both studies did not provide quantitative data on grain size or surface roughness, which are key parameters influencing the biological response, it is difficult to draw conclusions from these contradicting findings [262]. Taken together, many different roughness characteristics have a significant effect on the final cell response, including analysed timespan, roughness organization, geometry and scale, with each factor having its distinct influence on cell attachment.

#### Grain size and grain boundaries

**Figure 24.**
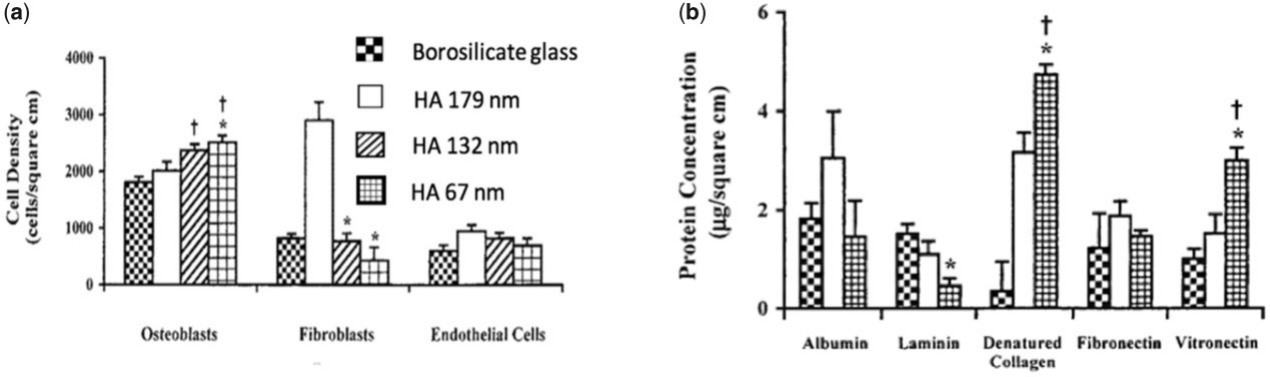
Cell attachment and protein adsorption on HA discs with varying grain sizes. (**a**) OB, fibroblast and EC adhesion on hydroxyapatite discs with various grain sizes. (**b**) Albumin, laminin, denatured collagen, FN and VN adsorption on hydroxyapatite discs. Obtained from Ref. [244]. Copyright © 2000 John Wiley & Sons, Inc.

For ceramics specifically, reduced grain size has been found to enhance protein and OB attachment, cell proliferation and gene expression [243, 264–267]. As grain size reduction and an increase in grain boundaries have been linked to increased surface roughness, improved cell attachment may result from the increased specific surface area [266]. As cell studies without protein-containing serum showed no correlation between grain size and cell attachment, it is believed that improved cell attachment on surfaces with smaller grains is a direct result of increased protein attachment [268]. Additionally, while Webster *et al.* showed enhanced cell attachment for OBs on HA surfaces with reducing grain size from 179 to 67 nm, the EC attachment decreased with reducing grain size [243, 265]. They linked this to the difference in specific protein attachment as a decrease in laminin adsorption, a protein that promotes EC attachment, and an increase in VN adsorption, a protein that stimulates OB cell attachment, was observed with decreasing grain size (Fig. 24) [243]. According to the authors, this is most likely a result of surface topography since VN, a small linear protein of 15 nm in length, may prefer surfaces with small grains and pores, while laminin, a much larger protein with a cruciform configuration being 70 nm in length and width, may prefer surfaces with larger grains and pores [243].

In contrast, a study carried out by Smith *et al.* on porous HA showed a reduction in OB cell attachment with decreasing grain size measured for incubation periods of up to 4 h. Proliferation, on the other hand, did significantly increase after 5 days of incubation on samples with smaller grains, which indicates its potential to allow more rapid OB growth rather than promoting initial attachment [269]. Another study carried out on dense HA also showed reduced cell adhesion on substrates with smaller grain sizes, but their surface roughness was found to decrease with decreasing grain size as well, which may have accounted for this contradiction [150].

Grain boundaries also play a significant role in the bioactivity of ceramics, with papers showing enhanced ion dissolution at these regions [151, 152]. The release of silicon into the ECM from Si-HA may aid the formation of a silicate network structure on the surface that can form a framework for protein and tissue organization, or the ions may directly improve the OB cell metabolism [33, 270]. Mladenovic, for example, found that silicon inhibits the RANK/RANKL/OPG signalling pathways related to osteoclast activation, and dissolution of this ion from Si-HA may therefore suppress bone resorption [31]. However, increased ion dissolution as a result of the smaller grain sizes on Si-HA may also simply increase the release of Ca^2+^ and ${\text{PO}}_{4}^{3-}$ ions into the ECM, resulting in enhanced formation of a calcium-phosphate layer [271]. These plausible explanations stress the need for a critical and thorough assessment of the influence of ion substitutions on the bioactivity of HA.

#### Surface modification and composition

A technique by which the bioactivity of scaffolds can be enhanced is known as surface modification. Ryan *et al.* [272] functionalized collagen scaffolds with copper-eluting bioactive glass and found enhanced osteogenesis and angiogenesis compared with non-functionalized collagen scaffolds and those functionalized with non-copper-doped bioactive glass. The authors ascribe this to the supposed ability of copper-ions to up-regulate VEGF production in, for example, MSCs and increase the proliferative capacity of ECs, as demonstrated in previous studies [260, 272]. Nevertheless, increasing the concentration of copper-doped bioactive glass from 0.1 mg Cu^2+^/ml to 0.3 mg Cu^2+^/ml reduced the angiogenic potential of the scaffolds, possibly due to the toxic effects on cells [272]. GFs such as VEGF and Ang1 can also be immobilized onto the collagen scaffolds which has been found to induce greater EC proliferation and vessel-like structure formation [273].

Heparin is another way by which the bioactivity of scaffolds can be improved as this is known to effectively bind with VEGF and other GFs. A recent study assessed the effect of heparin-loaded chitosan/HA scaffolds [274]. They observed a gradual heparin release from the scaffolds, which enhanced the angiogenic potential of the biomaterial when a low concentration of 28 µg heparin was applied [274]. As proposed by the authors, this effect stems from the binding of pro-angiogenic factors onto the heparin-loaded surface, which ensures a sustained availability of GFs throughout the process of tissue regeneration [274].

**Figure 25.**
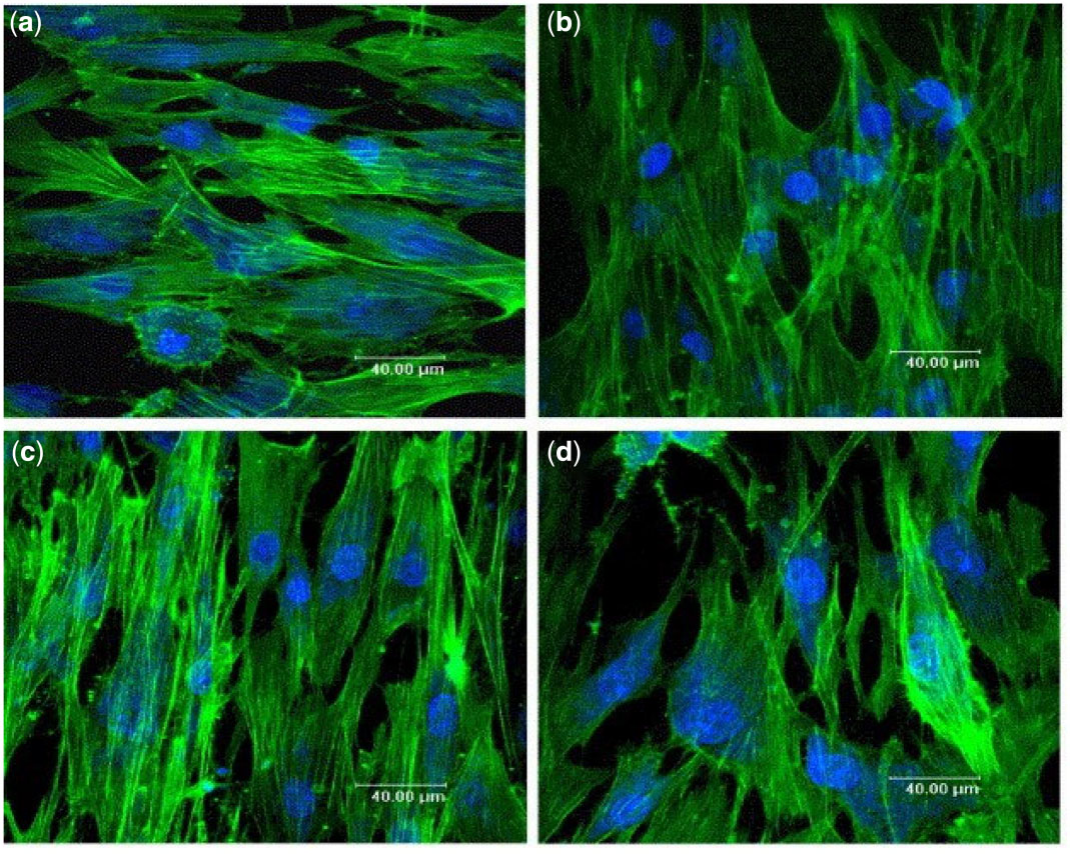
Immunostained images of OBs attached at Day 1 to (**a**) 0.8 wt% Si-HA, (**b**) 2.2 wt% Si-HA, (**c**) 4.9 wt% Si-HA and (**d**) HA. The nuclear DNA is stained blue, and the actin cytoskeleton is green. Images show enhanced OB proliferation on Si-HA with varying silicon content as compared with HA, and cells were well-oriented with a flattened cytoskeleton and distinct actin fibres [275]. Copyright ©2006, Elsevier B.V.

Dissolution of ions incorporated into the HA lattice, such as silicon, has also been suggested to positively influence OB behaviour and neovascularization. *In vitro* results obtained by Thian *et al.* [275] showed improved OB proliferation on Si-HA as compared with phase-pure HA, and cells were well-oriented with a flattened cytoskeleton and distinct actin fibres (Fig. 25). The authors also found improved mineral deposition on Si-HA, which they believe may have resulted from the formation of a silicate network on the surface of the biomaterial as a result of silicon ion dissolution [275]. Such a silicate network may also form a framework for protein and tissue organization [33, 270]. Additionally, an *in vivo* study carried out by Patel *et al.* [33] showed improved bone apposition on Si-HA as opposed to HA implants in New Zealand White rabbits at 23 Days after implantation. A quantitative assessment revealed a better bone ingrowth and on-growth, being the area of contact between implant and bone, as well as bone apposition rate on Si-HA implants. A study carried out by Magnaudeix *et al.* [36] assessed angiogenesis in chicken embryos and found Si-HA to be a good material for the conduction of blood vessels. Various other studies have reported on the effects of silicon on angiogenesis *in vitro* using different biomaterials such as akermanite and calcium silicate [37, 38]. These studies found that silicon appears to mainly influence the paracrine pathway between ECs and the supporting cell type by up-regulating key biomarkers for angiogenesis, such as VEGF, nitric oxide synthase and nitric oxide, that signal ECs to proliferate, migrate and form tubes [37, 38]. Patel *et al.* [33] suggested that improved bioactivity of Si-HA stemmed from the proposed role of silicon ions in up-regulating the OB cell metabolism, or from indirect effects related to changes in the physiological degradation properties as a consequence of the silicon substitution. While much remains uncertain about the mechanism behind the bioactivity of Si-HA, these results show the potential of silicon substitution as a way to improve the bioactivity of HA [33].

#### Surface charge

The surface potential of calcium phosphate discs may also play a crucial role in protein adsorption and cell attachment. Synthetic unsubstituted and silicon-substituted HA have an overall negative surface charge. Collagen has been reported to exhibit a negative surface charge close to zero at a physiological pH, but positively charged collagen scaffolds have also been observed [176, 276]. Collagen is also piezoelectric, meaning that it can generate electric signals in response to mechanical stress [177].

Many studies have focussed on protein adsorption to oppositely charged surfaces, as same-charged surfaces are known to generate electrostatic repulsion. However, to date, it is clear that proteins do also readily attach to same-charge surfaces, which may indicate that localized charges found on the various domains within the protein play a more crucial role. Proteins consist of multiple positive and negative charge groups that bind to specific ions available on the biomaterial surface, such as Ca^2+^, *PO*^3^_4_^−^ or Si^4+^, which may affect the conformation of the protein depending on their availability [277]. It is believed that the right orientation of the protein as well as its ability to unfold dictates whether a protein can or cannot attach to a charged surface [278, 279]. It is also well-known that more protein attachment does not always mean better attachment, as the quality of the protein layer is more important than the quantity [277]. Attachment to same-charged surfaces may also indicate that attachment cannot simply be narrowed down to the effects of electrostatics as other interactions such as hydrophobic interactions, hydrogen bonding and protein structure may play a more major part in the adsorption of proteins.

Studies also found charge heterogeneity across biomaterial surfaces which may impact the final protein and cell attachment [178, 180]. The variation in surface charge on a nanoscale is highly relevant when studying cell/protein–surface interactions, especially considering the nanoscale dimensions of serum proteins that generally exhibit a net negative charge. With the charge approaching zero towards the grain boundaries there may be less repulsion between the overall charge of the proteins and the surface at these regions, or the heterogeneity may influence the protein conformation and orientation [178]. Additionally, assessment of Si-HA surfaces, carried out by the same group, revealed that silicon substitution increases the nanoscale electrostatic, van der Waals and adhesive interactions. The authors hypothesized that the increased adhesive forces on these surfaces may overcome the increased repulsion between the more negative surface charge and various proteins and OBs, having a negatively charged cell membrane [180].

While cell attachment and morphology appear to be modulated by the way proteins respond to the surface charge, it has also been found to be influenced by the surface potential directly [277, 280]. In a serum-free medium, positively charged surfaces were found to promote attachment of rat marrow stromal cells, possibly due to the net negative charge of cell membranes under physiological conditions, but were at the same time found to suppress cell spreading and differentiation [280]. George *et al.* [276] proposed that the positive charge measured on their honeycomb collagen scaffolds could be beneficial to MSC attachment due to the negative charge of integrins on the cell membrane.

A study carried out on carbon nanoparticles (CNPs) grafted on high-density polyethylene, found improved proliferation and adhesion of vascular smooth muscle cells on nanoparticle grafted surfaces functionalized using amine-amide groups [281]. Amine-amide functionalization changes the surface charge of CNPs from negative to positive, which the authors believe may have had a positive effect on the attachment of cells due to their negatively charged membrane [281]. However, other factors such as a strong increase in hydrophilicity may have also played a role. Overall, again, due to the intertwined nature of various surface properties, the precise influence of surface charge on cell attachment remains unclear and should be investigated further.

Taken together, much remains unknown about the biological response to biomaterials, and the generalizability of published research is problematic due to the complex nature of protein and cell attachment and the numerous intertwined surface properties that cannot be individually assessed. Throughout the years, various patterns have been extracted, which are summarized in Table 3, but continued research is crucial to improve the understanding of the mechanisms in more detail to enable successful advancement in the field of tissue engineering [148–150].

**Table 3.** Summary of the cell/protein and surface interactions, as reported in literature

Property	Protein response	Cell response	Refs
Wettability	Hydrophobic surfaces promote stronger association, but hydrophilic surfaces are suggested to induce a better quality of the protein layer. VEGF domains on FN exposed only on hydrophilic surfaces.	Lower attachment to hydrophobic surfaces.	[120, 185, 223, 240, 241, 246]
Roughness	Proteins respond to nanoscale features. Roughened surfaces entrap more proteins and expose more surface area for attachment.	Increased surface area allows for more binding sites. Increased cell attachment was detected on organized rough surfaces as opposed to disorganized surfaces. Cells require a certain roughness ratio to allow for appropriate positioning of the integrin clusters necessary for anchorage to the surface.	[226, 250–254, 256, 257, 262, 263]
Geometry	Grooves increase the surface area available for attachment.	Grooves increase the surface area available for attachment and have been found to align cells. Vascular smooth muscle cells and ECs favour aligned grooves	[258]
Grain size	Effect depends on protein type (mainly protein size) and the impact of grains on the surface roughness. The reduction in grain size is often associated with increased surface roughness which generally results in better attachment.	Effect depends on cell type and the impact of grains on the surface roughness.	[265, 266]
Grain boundaries	Increased number of grain boundaries may increase the surface roughness increasing the surface area exposed to proteins.	Increased ion dissolution found at grain boundaries is associated with better osseointegration.	[151, 152]
Surface modification and composition	Incorporation of silicon ions into the HA lattice may improve protein attachment.	Loading surfaces with bioactive agents such as heparin can improve cell osteogenesis and angiogenesis.	[274, 275]
Charge	Protein orientation and unfolding as a result of the localized protein and surface charge are believed to dictate the quality of the attachment.	Positive surfaces may attract cells due to their negative cell membrane but the overall cell attachment and behaviour appears to be more strongly modulated by the protein response to the surface charge.	[276, 277, 280]

### Structural effects on angiogenesis

The architecture of tissue-engineered scaffolds also has an impact on vascularization. The effect of various structural parameters of scaffolds has been assessed regarding their influence on vascular network formation and vessel in growth through guiding cellular distribution as well as organization. Pore size, the diameter of individual cavities, and porosity, the ratio of the cavity to scaffold volume of fabricated constructs can have a significant impact on angiogenesis. Several studies have established that a sufficient increase in the pore size of scaffolds can significantly enhance vascular network formation [282–285]. Druecke *et al.* [282] applied a systematic approach to investigate the optimal pore size of poly(ether ester) block-copolymer constructs for inducing an efficient angiogenic response. Scaffolds with different pore diameters, categorized as small (20–75 µm), medium (75–212 µm) and large (250–300 µm), were implanted into the dorsal skinfold chamber of mice and were monitored together with the surrounding tissue for up to 20 days [282]. A significantly larger vessel diameter at the centre of the scaffolds was recorded for scaffolds with large pores (13.8 ± 0.8 µm) as opposed to small pores (10.11 ± 0.7 µm), and little difference was observed between the medium and large pore size scaffolds [282]. Another *in vivo* study explored the relationship between various macropore sizes, ranging from 300 to 700 µm, and angiogenesis within β-TCP scaffolds [283]. They observed that an increase in pore size resulted in greater neovascularization. Specifically, pore diameters below a 400 µm limit exhibited reduced neovascularization resulting in smaller blood vessel diameters, which is expected as pores provide the space for vessel growth [283]. The observed increase in fibrous tissue ingrowth in pores with decreasing pore diameters, occupying pore space and consequently reducing the remaining space for vascular ingrowth, accounts for an even further decrease in the vessel diameter [283]. Another study contradicted this, showing reduced neovascularization and increased ingrowth of fibrous tissue on scaffolds with larger pores (≈90–160 µm) [286]. However, only average pore sizes were reported in this study and a broad pore size distribution was detected, making it difficult to draw a firm conclusion regarding the optimal pore size [286].

Furthermore, a computer-based 3D model was developed by Mehdizadeh *et al.* [284] to investigate the effect of architectural parameters of porous scaffolds on sprouting angiogenesis. The influence of pore size, interconnectivity, and porosity of scaffolds on vascularization was investigated for a rapid prediction of angiogenesis behaviour of different *in vivo* and *in vitro* experiments [284]. While the average invasion depth (AID) in scaffolds with pore sizes of 275 and 400 µm was high with blood vessels reaching the core of the constructs, the AID of scaffolds with a pore diameter of 150 µm was low with vessels reaching less than half of the scaffold depth [284]. Interestingly, although increasing interconnectivity of homogenous scaffolds simulated for 6 weeks had little effect on scaffolds with smaller pores, it had a great impact on those with larger pores resulting in a significant increase in AID and blood vessel density [284]. In a more recent study, angiogenesis in β-TCP scaffolds with similar macropore sizes (300–400 µm) but different interconnectivity ranging from 100 to 150 µm was investigated [285]. Upon 4 weeks of implantation, a significantly larger blood vessel density was observed in scaffolds with the highest interconnectivity of 150 µm compared with all other samples, with an even larger difference observed after 12 weeks post-implantation [285]. More strikingly, although the area and volume percentage of blood vessels were significantly lower in the scaffolds with lower interconnectivity, almost no difference in mean vessel diameter was observed, suggesting that the increased area and volume percentage are a result of sprouting angiogenesis, accounting for a relative increase in the number of blood vessels [285].

Pore shape, pore morphology and above all the structural orientation of a biomaterial are believed to affect the growth of microvessels on these materials. In recently published work, highly aligned decellularized human dermal fibroblast sheets were used to investigate the impact of topographical features on microvasculature network formation using a co-culture of ECs and MSCs [287]. Structural alignment not only improved the density, maturity and structure of vessels compared with the control group with non-aligned features, but it also improved both pre-angiogenic GF secretion and ECM remodelling, indicating well-established cellular cross-talk between the two cell populations [287]. In an *in vivo* study, the effect of different pore architectures, concave and convex pore shapes, on angiogenesis as part of ectopic bone formation was investigated on HA scaffolds with high porosity and interconnectivity [288]. After a month of implantation, the vascular density appeared to be higher in convex HA scaffolds but was exceeded by that of concave scaffolds upon 6-month post-implantation [288]. The authors suggested that while the honeycomb-like pores of convex scaffolds are more attractive for both calcium layer deposition and vessel formation at early stages, it is the concave pore structures that are more conducive to apatite layer formation as well as longitudinal vessel formation for longer implantation periods [288].

Overall, several different architectural features and chemical compositions have been identified to have an impact on neovascularization as they influence cellular migration and interaction within porous scaffolds. Nevertheless, to date, most studies on the bioactivity of bioceramics and collagen scaffolds have been carried out in *in vitro* monocultures. More complex *in vitro* co-culture studies using OBs and ECs could offer a closer approximation of the *in vivo* environment in a controlled setting by simulating the simultaneous bone and blood vessel growth. Therefore, continued research on the effects of structural properties of 3D constructs and their chemical make-up on the biological response in co-culture could provide more insight into *in vivo* mechanisms and is deemed to be essential for understanding the development of mature, dense and intact vessels, which is important for a successful anastomosis upon implantation [289].

## Conclusion

To summarize, synthetic polymers such as collagen and calcium-phosphates are widely used as biomaterials for BTE due to their chemical resemblance to the organic and inorganic components of bone, respectively. While they are promising, various drawbacks of these materials such as weak mechanical properties and low thermal stability for synthetic polymers and low degradation rate and brittleness for calcium-phosphates still need to be overcome. Composites of these materials have been suggested which benefit from the elasticity of polymers and the relative stiffness of the calcium phosphates. While the material properties have continued to improve over the years, one major challenge in BTE remains the simultaneous formation of a vascular network and bone tissue. As vascularization provides nutrients and removes waste products, it is crucial for cell survival within a scaffold. Various strategies exist to achieve neovascularization in scaffolds such as GF-releasing scaffolds, cell-based strategies and prevascularization. Although pro-angiogenic GFs are important for mimicking natural tissue neovascularization, uncertainty remains regarding the dose-effect ratio, the suitable factors and delivery vehicles and appropriate timing. Cell-based strategies have shown improved angiogenic potential compared with the GF-releasing strategy, but due to the size limitation of this strategy at this stage, it cannot induce successful vascularization in scaffolds of clinically relevant size. Apart from these limitations, GF-releasing and cell-based strategies are also time-consuming as they rely on the ingrowth of host tissue for vascularization. Prevascularization, on the other hand, involves scaffolds with pre-formed capillaries accommodating the integration of the scaffold into the host tissue. Based on the current literature, *in vitro* pre-culturing of scaffolds using either microvessel fractions or cells in co-culture shows promising results regarding ingrowth, density and maturation of vessels and could serve as an appropriate alternative to other strategies with a co-culture of ECs with supporting cells being studied widely. Gaining fundamental knowledge of the mechanism behind the bioactivity of biomaterials and understanding the contribution of scaffold features to the biological response can provide an invaluable basis for future optimization studies regarding the vascularization of large constructs. Although *in vitro* mono-culture studies can serve as a useful tool to provide initial insight into the cell–material interactions, cellular cross-talk within a more complex system may significantly alter the outcome, demanding additional characterization of cells using co-culture studies. Optimization of scaffold characteristics and co-culture parameters is challenging but necessary for engineering vascularized tissue grafts. In particular, the cell ratio and architectural features have been found to play a fundamental role in obtaining the desired physiological equilibrium. Several studies have optimized co-culture parameters on both polymer and calcium phosphate scaffolds and have achieved successful formation of a microcapillary network *in vitro*. To date, various trends can be extracted from literature, but a systematic understanding of how architectural features, biochemical properties and co-culture parameters influence angiogenesis for bone tissue regeneration is still lacking despite its importance for scaffold functionality and medical applications. In terms of surface properties, it has been observed that more hydrophilic surfaces enhanced cell attachment and improved the quality of the protein layer, by, for example, exposing VEGF domains on FN. Increased surface roughness was found to be associated with better protein and cell attachment and vascular smooth muscle cells and ECs favour grooved surfaces. A larger number of grain boundaries induces increased ion dissolution resulting in improved biological response. Lastly, the addition of bioactive agents such as heparin have been observed to improve osteogenesis and angiogenesis and heterogeneity in surface charge has been found to dictate the protein unfolding which subsequently affects the cell response. In terms of architectural features, studies found that pore size and porosity of fabricated constructs strongly influence the angiogenic response of the construct. Generally, a larger pore size was found to be associated with the formation of larger vessels. Another study found an increased invasion depth of vessels in scaffolds with large pores and increasing the interconnectivity improved the invasion depth even further. Increased interconnectivity in scaffolds with smaller pores, on the other hand, did not appear to have the same beneficial effect on the vessel invasion. Additionally, increased interconnectivity has been associated with improved sprouting angiogenesis resulting in a higher vessel density insight the scaffolds. Another important structural property assessed in literature is the alignment of the pores, with evidence showing that structural alignment improved the density, maturity and structure of vessels and it also increased both pre-angiogenic GF secretion and ECM remodelling compared with non-aligned features. While there appears to be a consensus in the literature regarding the effect of surface properties and architectural features on the biological response, various studies do indicate the existence of an optimum range of these properties which can differ depending on the material, protein and cell types investigated, complicating the generalization of these trends. Taken together, continued *in vitro* research in co-culture is crucial to gain a better understanding of the effect of material properties on cell behaviour in a complex *in vivo* setting, and any change in the environment, including the use of different cell or protein types, should demand new characterization to determine the optimal surface properties and architectural features of the investigated scaffold.
